# The need to approximate the use-case in clinical machine learning

**DOI:** 10.1093/gigascience/gix019

**Published:** 2017-03-15

**Authors:** Sohrab Saeb, Luca Lonini, Arun Jayaraman, David C. Mohr, Konrad P. Kording

**Affiliations:** 1 Department of Preventive Medicine, Northwestern University, 10th floor, Rubloff Bldg, 750 N Lake Shore Dr, Chicago, IL 60611, USA; 2 Department of Physical Medicine and Rehabilitation, Northwestern University, 345 E Superior St, Suite 1479, Chicago, IL 60611, USA; 3 Shirley Ryan Ability Lab, Room 1401, 11th Floor, 355 E Erie St., Chicago, IL 60611, USA

**Keywords:** Machine learning, cross-validation, clinical outcomes, rehabilitation outcomes, prediction accuracy, diagnosis, smartphones, wearable technology

## Abstract

The availability of smartphone and wearable sensor technology is leading to a rapid accumulation of human subject data, and machine learning is emerging as a technique to map those data into clinical predictions. As machine learning algorithms are increasingly used to support clinical decision making, it is vital to reliably quantify their prediction accuracy. Cross-validation (CV) is the standard approach where the accuracy of such algorithms is evaluated on part of the data the algorithm has not seen during training. However, for this procedure to be meaningful, the relationship between the training and the validation set should mimic the relationship between the training set and the dataset expected for the clinical use. Here we compared two popular CV methods: record-wise and subject-wise. While the subject-wise method mirrors the clinically relevant use-case scenario of diagnosis in newly recruited subjects, the record-wise strategy has no such interpretation. Using both a publicly available dataset and a simulation, we found that record-wise CV often massively overestimates the prediction accuracy of the algorithms. We also conducted a systematic review of the relevant literature, and found that this overly optimistic method was used by almost half of the retrieved studies that used accelerometers, wearable sensors, or smartphones to predict clinical outcomes. As we move towards an era of machine learning-based diagnosis and treatment, using proper methods to evaluate their accuracy is crucial, as inaccurate results can mislead both clinicians and data scientists.

## Introduction

Machine learning has evolved as the branch of artificial intelligence that studies how to solve tasks by learning from examples rather than being explicitly programmed. Machine learning has grown massively over the past decades, with countless applications in technology, marketing, and science [1]. Almost every smartphone nowadays includes speech recognition. Social media and e-commerce websites filter contents and recommend products based on the user's interests, and scientific data, from astrophysics [2] to neurophysiology [3] and medicine [4], are analyzed using machine learning algorithms.

In medicine, a great hope for machine learning is to automatically detect or predict disease states as well as assist doctors in diagnosis, using data collected by phones and wearable sensors. A driving factor is that people carry these devices with them most of the time, and thus a growing amount of daily life data, such as physical activities [5, 6], is becoming available. Indeed, an increasing number of studies apply machine learning to the data collected from these devices for clinical prediction purposes. Examples include detecting cardiovascular diseases [7], falls [8], measuring rehabilitation outcomes in stroke and amputees [9–11], monitoring Parkinson's disease (PD) symptoms [12–14], and detecting depression [15, 16].

The majority of machine learning algorithms used for clinical predictions use the supervised learning approach, which can be summarized in the following steps: first, a set of features is computed from the raw data. These features are typically engineered depending on the specific application; e.g., one feature could be the maximum heart rate in a given time interval. Features are then fed into a machine learning classifier, the parameters of which are adjusted to map each input data point (feature vector or *record*) to its corresponding label, e.g., “healthy”. Once the classifier is trained on enough data, it can be used to perform predictions on new subjects using their features; e.g., do their features predict that they are healthy?

A crucial stage of this process is to assess the prediction accuracy of the trained machine learning algorithm. The standard approach is to use cross-validation (CV) [17], where the data is split into training and test subsets. Splitting data into training and test subsets can be done using various methods, such as leave-one-out, leave-*p*-out, *k*-fold, and Monte-Carlo sampling [18]. The classifier is trained on the training set, while its accuracy is measured on the test set. The aim of this process is to assess the ability of the machine learning algorithm to generalize to new data.

For CV to be valid, training and test sets need to be independent [30]. The definition of independence, however, depends on the use-case scenario. If the use-case is *diagnosis*, i.e., we want to develop *global models* that can be used for new subjects, CV must be subject-wise, meaning that the training and test sets contain records from different subjects. On the other hand, if the goal is *prognosis*, i.e., we want *personal models* that can predict future clinical states of a given subject, we can split the data based on time, so that training and test sets contain records of the same subject from different times. In more complex scenarios, we may want to generalize from one clinical site to another, in which case CV needs to be across these sites [19]. Therefore, the choice of the appropriate CV method depends on the application.

**Figure 1:**
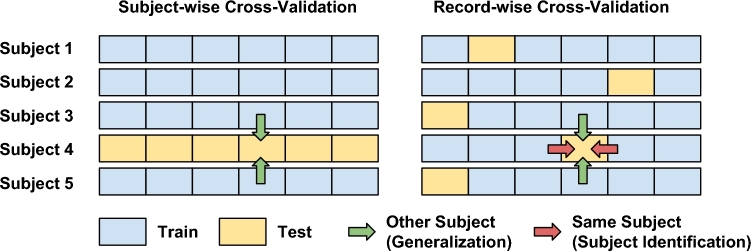
A visualization of subject-wise (**A**) and record-wise (**B**) CV for a diagnosis scenario. Data is split into training (blue) and test (yellow) sets to evaluate the performance of machine learning algorithms. Each box represents a record from each subject. While subject-wise CV only uses data from other subjects (green arrows), record-wise in addition uses data from the same test subject (red arrows) to predict its state. The problem is, that if the algorithm can (implicitly) detect the identity of the person based on the features, it can automatically also “diagnose” the disease.

In a diagnosis scenario (Fig. 1), we want to build a model that can generalize to new subjects; thus we must use subject-wise CV (Fig. 1A). Nevertheless, one can employ *record-wise* CV, which randomly splits data into training and test sets regardless of which subjects they belong to (Fig. 1B). Therefore, records from the same subject are present in both training and test sets. In this way, the machine learning algorithm can find an association between unique features of a subject (e.g., walking speed) and their clinical state, which automatically improves its prediction accuracy on their test data. As a consequence, the record-wise CV method can overestimate the prediction accuracy of the algorithm.

The following hypothetical example clarifies how using record-wise CV in a diagnosis problem is misleading. Imagine we recruit four subjects, two healthy and two affected by PD. We have a machine learning algorithm that estimates if a person has PD based on their walking speed. Let our two healthy subjects have constant walking speeds of 1 meter per second (m/s) and 0.4 m/s, and our two PD patients 0.6 m/s and 0.2 m/s. If we do subject-wise CV, we will be unable to predict the performance of the slow healthy subject as well as the fast PD subject, resulting in a prediction accuracy of roughly 50%. Now for record-wise CV, let us have 10 records for each subject. To predict the first of the 10 measurements of the fast healthy subject we would also use the other 9 measurements of that same subject. We would thus be able to certainly conclude that this subject is healthy. The same would be true for the slow healthy subject. After all, none of the PD patients has a walking speed of 0.4 m/s. Subject identification, which we know to be relatively easy, thus replaces disease recognition, which we know to be hard. As such, record-wise CV would give us a 100% accuracy, which is clearly not supported by the data, and therefore the algorithm will not generalize.

The aim of this paper is (i) to demonstrate the potential bias caused by using inappropriate CV methods for clinical prediction applications, and (ii) to examine how widespread the problem is. We examine the first aim by showing that record-wise and subject-wise CV yield dramatically different results, with record-wise CV massively overestimating the accuracy. We demonstrate this by using a publicly available dataset on human activity recognition, as well as a simulation that shows how subject-specific and disease-specific factors interact. For the second aim, we conduct a systematic literature review to quantify the prevalence of this problem in studies that use smartphone and wearable sensor data to predict clinical and rehabilitation outcomes. We report the proportion of papers using record-wise CV against those using subject-wise CV, along with their classification accuracies and number of citations.

## Human activity recognition

### Data description and methods

First, we evaluated how record-wise and subject-wise CV would be different in a real dataset. We chose a publicly available human activity recognition dataset [20, 21], which contained recordings of 30 subjects performing 6 activities: sitting, standing, walking, stair climbing up/down, and laying down. Data consisted of recordings from the accelerometer and gyroscope sensors of a smartphone carried by the subjects. Each data record was a vector of 561 features computed from the sensors signal over a time window of 2.56 s. The dataset contained a total of 10 299 records, with an average of 343 records per subject and an approximately equal number of records per activity.

We used random forests [22] in MATLAB R2015a for classification. A random forest is an ensemble of decision trees, with each tree providing a prediction about the class of the input data. The forest's prediction is determined by aggregating the predictions of individual trees. Each tree in a random forest only sees a subset of features and a subset of input data samples. A random forest, thus, has fewer parameters to tune, which makes it less prone to overfitting and a better candidate for generalization to unseen data. We also found random forests to perform well in our previous activity recognition study [23]. Therefore, random forests were an appropriate choice for this study.

For each of the record-wise and subject-wise methods, we used 2, 10, or 30 subjects, and *k*-fold CV^1^ with *k* = 2, 10, or 30. For record-wise, data was randomly split into CV folds regardless of which subject it came from. For subject-wise, we split data by subjects such that training and test folds contained records from different subjects. In both methods, the classifier was trained on all but one fold and tested on the remaining fold. The number of trees for the random forest classifier was set to 50, which was optimized based on the out of bag error [22]. We repeated the training procedure 100 times, such that new subjects and folds were randomly generated in each repetition.

### Analyses

**Figure 2:**
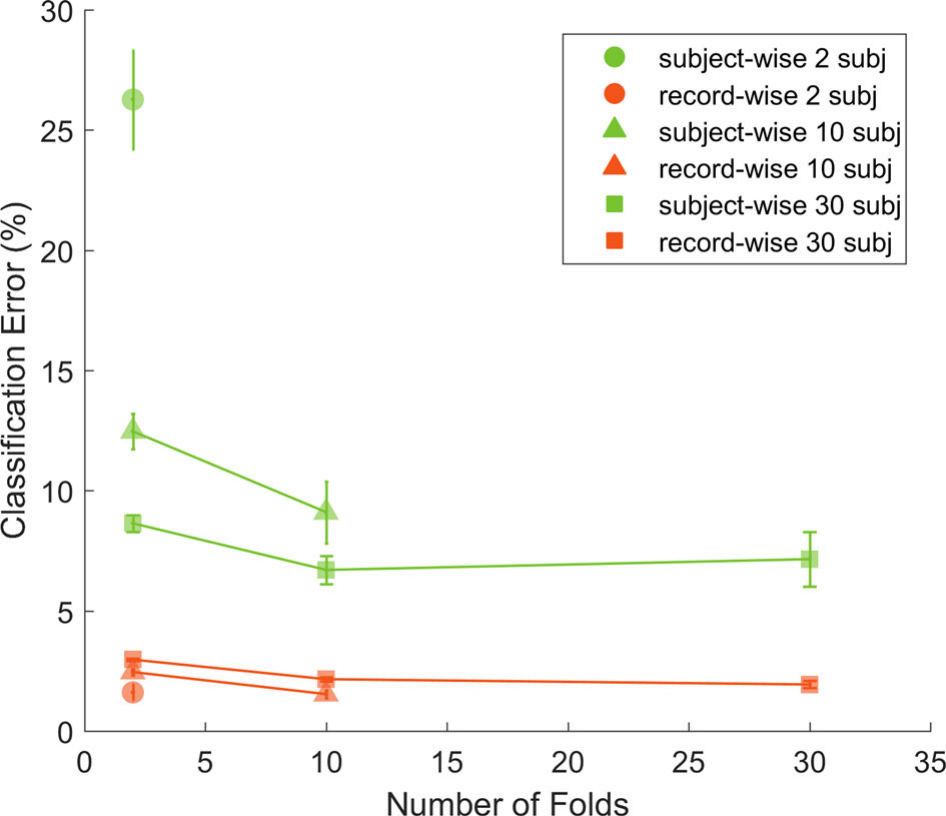
Effect of subject-wise and record-wise CV on the classification error for the UCI activity recognition dataset. As the number of folds (x-axis) used to perform CV increases, the error tends to decrease. Similarly, performance improves when the number of subjects increases (symbols denote total number of subjects used to train and test the classifier). Record-wise CV significantly inflates the predicted accuracy (orange) as compared to subject-wise CV (green). Error bars indicate 95% CIs.

We started by evaluating the performance of subject-wise CV on the activity recognition dataset. When using 2 folds and 2 subjects only, the error rate started at a value of 27% and, as the number of subjects increased to 30, it decreased significantly and reached 9% (Fig. 2). Similarly, as the number of folds increased, i.e., data from more subjects were used for training the classifier, the error rate decreased and leveled around 7% with 30 folds (Fig. 2, green lines).

We then trained the classifier using record-wise CV and used the same procedure to assess how the error changed as a function of number of subjects and folds. Interestingly, the classification error already started at a value of 2% when using data from only two subjects, and did not significantly change when either number of subjects or folds increased (Fig. 2, orange lines). Therefore, regardless of the amount of data used for training the classifier, record-wise CV significantly overestimated the classification accuracy on this dataset.

## Simulated clinical prediction

### Data description and methods

In the second part of our study, we generated a simulated dataset to find out which properties of human subject data make the performance of subject-wise and record-wise CV methods different. Specifically, we were interested in two properties: *cross-subject variability* and *within-subject variability*. Cross-subject variability is the variability in data that is observed across the subjects. In a diagnosis scenario, this property captures the effect of the disease, or the features that distinguish healthy subjects from sick. Within-subject variability represents the variability observed when multiple samples are recorded from a single subject. In clinical data, this property is usually related to the changes in the disease trajectory, as well as changes in the physiology of the subjects.

We used a generative model to create the simulated dataset. This model combined cross-subject and within-subject variabilities to generate an observation as the following:

(1) \begin{equation*} {y_{s,r,n}} = a{\beta _s} + b{u_{s,n}} + c{v_{s,r,n}} + d{\varepsilon _n} \end{equation*}

where *y*_*s, r, n*_ is the observed value for feature *n* (*n* ε {1, 2, …, *N*}) in record *r* (*r* ε {1, 2, …, *R*}) collected from subject *s* (*s* ε {1, 2, …, *S*}). *N, R*, and *S* are the number of features, the number of records per subject, and the total number of subjects, respectively. β_*s*_ encodes disease effects, or fixed effects, with 1 for patients and −1 for healthy subjects. ${u_{s,n}}\sim{\mathcal N}( {0,1} )$ accounts for the random cross-subject variability, or random effects, and ${v_{s,n,r}}\sim\mathcal{N}( {0,1} )$ represents the within-subject variability. Finally, ${\varepsilon _n}\sim \mathcal{N}( {0,1} )$ is the population-level feature-generating process. *a, b, c*, and *d* are constant parameters, where *b* and *c* control the proportions of cross- and within-subject variability, respectively.

We generated datasets with variable number of subjects, from 4 to 32. These numbers were within the range used in the reviewed studies, as well as in the activity recognition dataset. In each simulated dataset, half of the subjects were set to diseased (β_*s*_ = 1) and half were healthy (β_*s*_ = −1). We used *N* = 10 features and *R* = 100 records for each subject. We set *a* = 0.5, accounting for a disease effect size of (1−[−1]) x 0.5 = 1, and *d* = 0.1. We varied *b* and *c* between 0 to 2 with increments of 0.1 to test the effect of cross- and within- subject variability on the CV results.

Similar to the activity recognition dataset, we trained and tested random forests on the simulated dataset, using both record-wise and subject-wise methods, to predict whether a record came from a patient (β_*s*_ = 1) or a healthy subject (β_*s*_ = − 1). For record-wise, we randomly split the dataset into 50% training and 50% test, regardless of the subject IDs. For subject-wise, we did the same but we ensured that same subjects were not present in both training and test sets. For each CV method and each value of *b* and *c*, we measured the classification error on the test set. The simulation was repeated 10 times for each number of subjects and each value of *b* and *c*, and the average prediction error across the repetitions was calculated. The generative model code is available for download as detailed in the section “Availability of Supporting Data and Materials.”

Finally, we wanted to see how the prediction errors evaluated by subject-wise and record-wise CV methods compared to the true prediction error. To find the true prediction error, we generated an additional dataset of 10 subjects, which were not used for CV, using the same generative model (Equation 1). We calculated the true prediction error by evaluating the performance of each of the trained classifiers on this new dataset. In this way, in addition to evaluating the difference between subject-wise and record-wise prediction errors, we could see which was closer to the true prediction error.

### Analyses

**Figure 3:**
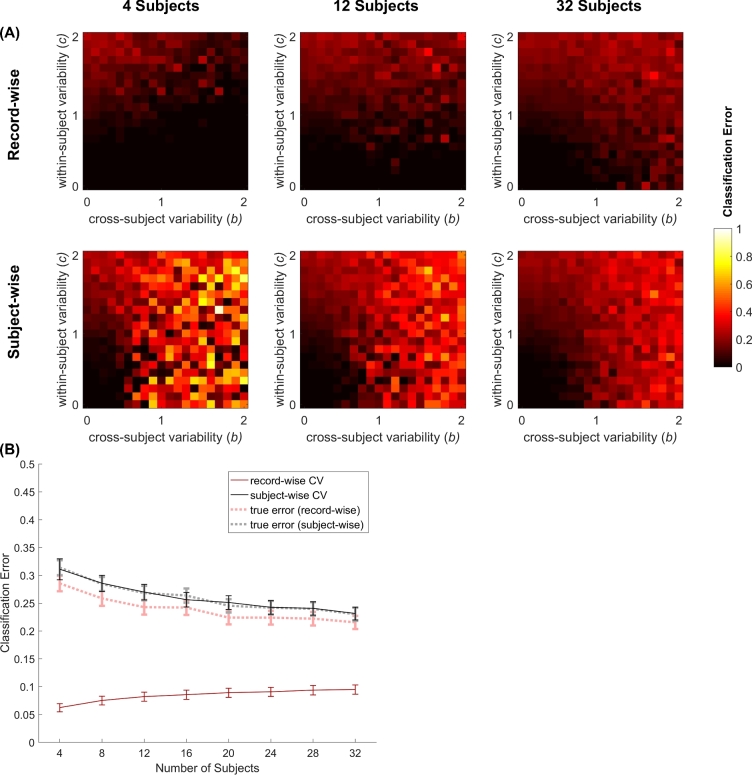
Classification error in a simulated dataset for record-wise and subject-wise CV methods, as a function of cross-subject (*b*) and within-subject (*c*) variability (**A**), and number of subjects (**B**). The number of features is set to 10. (A) Each column shows the classification error for both CV methods as a function of *b* and *c* for a given number of subjects (4, 12, and 32). Brighter colors indicate higher classification error values (black = 0; white = 1). (B) The mean and the standard deviation of classification error, across the values of *b* and *c*, for subject-wise and record-wise methods, as a function of number of subjects.

We quantified how cross-subject variability (*b*) and within-subject variability (*c*) contributed to the classification errors in either of the two CV methods (see Equation 1). As in the activity recognition task, we used a random forest classifier. Fig. 3A shows how classification error changed with *b* and *c*, for 4, 12, and 32 subjects. In all scenarios, for small values of *b* and *c* the classification error was small. This is because when both within- and cross-subject variabilities are low, the disease effect dominates the feature values, helping the classifier to distinguish between healthy and diseased subjects. For higher values of *c*, classification errors were higher, especially for the subject-wise method.

Increasing the cross-subject variability (*b*) alone did not increase the classification error for the record-wise method, when the number of subjects was small (top left panel). Indeed, in the record-wise CV, the classifier was already informed about *b* by having samples from all or most of the subjects in its training set. For the subject-wise method, on the other hand, increasing *b* dramatically increased the classification error (bottom left panel). Nevertheless, when more subjects were used, the classification error increased in both CV methods, but remained lower for the record-wise method (top right versus bottom right panel).

Overall, as shown in Fig. 3B, record-wise CV underestimated the classification error relative to the subject-wise CV method. This was further confirmed by comparing the prediction error yielded by each CV method with the true error, shown by dashed lines in Fig. 3B, which was computed by testing each trained model on a new dataset that was not used for CV. The prediction error estimated by subject-wise CV (black line) was closer to the true error, while record-wise CV (red line) massively underestimated it. In addition, subject-wise CV errors closely followed the true classification error as the number of training subjects changed.

The difference between the record-wise and subject-wise errors was largest when the number of subjects was small, and gradually decreased as we increased the number of subjects. This is because as we increase the number of subjects, it becomes harder for the record-wise classifier to identify subjects based on their feature values, thereby losing its advantage over the subject-wise method. Nevertheless, even for relatively large number of subjects, record-wise CV led to an underestimated classification error, while the subject-wise CV had a significantly closer estimate of the true performance of the algorithm.

## Systematic review

**Figure 4:**
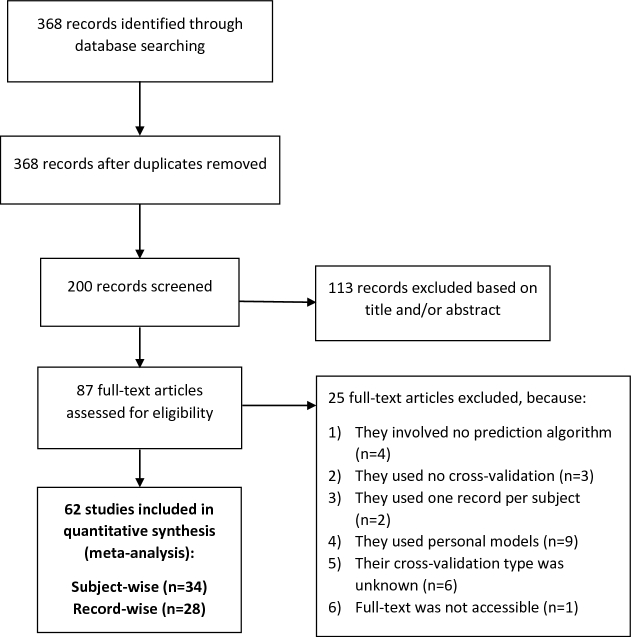
PRISMA flowchart.

In the last part of our study, we systematically reviewed the literature to find out how many published studies used record-wise versus subject-wise CV. We specifically looked for the studies which used both machine learning and smartphone or wearable sensor technology for clinical predictions. This process had three main steps: (i) searching for relevant papers; (ii) excluding papers which did not meet our eligibility criteria; and (iii) determining the CV type used in each paper. This process was consistent with PRISMA guidelines (Fig. 4). The papers were reviewed and analyzed by two authors (SS, LL), and a total of four discrepancies were resolved by consensus.

### Search strategy

We wanted to find the studies which contained at least one of the following keywords in their title: “wearable,” “smartphone,” or “accelerometer.” In addition, to account for the clinical applicability, the papers had to have one of the following keywords in their text: “diagnosis,” “disease,” or “rehabilitation.” Finally, the papers had to contain “cross-validation” at least once, and be published after 2010. To account for these search criteria, we used Google Scholar [24] with the following search query:

*(intitle: wearable OR intitle:smartphone OR intitle:smartphones OR intitle:accelerometer) AND (diagnosis OR disease OR rehabilitation) “Cross-Validation” (from 2010)*

### Eligibility criteria

1) Peer-reviewed studies published in English.
2) Studies which used a machine learning technique to predict a clinical or rehabilitation outcome.
3) Studies which used CV to assess the performance of their algorithms.
4) Studies which used more than one data point (record) for each subject. In fact, when there is only one record per subject, record-wise CV and subject-wise CV are the same.
5) Studies which did not use *personal* models, which are trained and tested on each individual subject separately.
6) Studies which were not review of the literature or book chapter.
7) Studies for which the CV type was not “unknown” (see Determining the CV type).

### Determining the CV type

After finding the papers which met our eligibility criteria, we grouped them into the ones which used record-wise CV and the ones that used subject-wise CV. We assigned a paper to the subject-wise group if one or more of the following conditions were satisfied:

1) The authors used the term “subject-wise” or “leave-one-subject-out” when explaining their CV strategy.
2) The authors mentioned that they tested their algorithms on subjects that were not included in the training set.
3) We did not find any overlap between the subject IDs in training and test datasets, where subject IDs were provided.

We assigned a paper to the record-wise group if one or more of the following conditions were satisfied:

1) The number of CV folds was greater than the number of subjects.
2) The authors mentioned that they randomly split the whole dataset into training and test subsets.
3) We found an overlap between the subject IDs in the training and test datasets, where subject IDs were provided.

If none of the 6 conditions above were satisfied, we labeled the CV type as “unknown”.

### Extracting other metrics

We also wanted to see if subject-wise and record-wise CV studies were different in the number of citations they received, as well as in their reported classification accuracy. For the number of citations, we used the information provided by Google Scholar. For the accuracy, however, we analyzed the full-text papers. Since papers used different metrics to report the classification accuracy of their algorithms, we used the following rules to find their accuracies:

1) Where a single accuracy or classification error value was reported, we directly used that.
2) Where multiple accuracies were reported for different conditions or classes, we used the average accuracy.
3) Where accuracies were reported for different types of classifiers or feature sets, we used the highest accuracy.
4) Where sensitivity and specificity were reported and their difference was <2%, we used their average as accuracy. This is supported by the fact that accuracy is bounded by sensitivity and specificity.^2^
5) Where F1-score was reported, or precision and recall were reported from which we could calculate the F1-score, we used it instead of the accuracy.
6) We did not extract the classification accuracy from the papers which only reported root-mean-square error (RMSE), normalized RMSE (NRMSE), or similar metrics.

### Analyses

A total of 369 papers were initially retrieved from Google Scholar. Of these, we screened the first 200 papers. 113 papers were excluded upon the review of the title and the abstract because they: (i) reported studies that were not related to any clinical or rehabilitation outcomes (n = 98), (ii) were review of the literature or book chapters (n = 10), (iii) were dissertation reports (n = 4), or (iv) used personal models (n = 1). We retrieved the full text of the remaining 87 papers. Of these, we excluded 25 more papers, based on the criteria detailed in Fig. 4. We included the remaining 62 papers in our systematic review. We found that 28 of these papers used record-wise while 34 used subject-wise CV. Therefore, about 45% of the reviewed papers used the record-wise CV method for estimating the performance of their global models.

**Figure 5:**
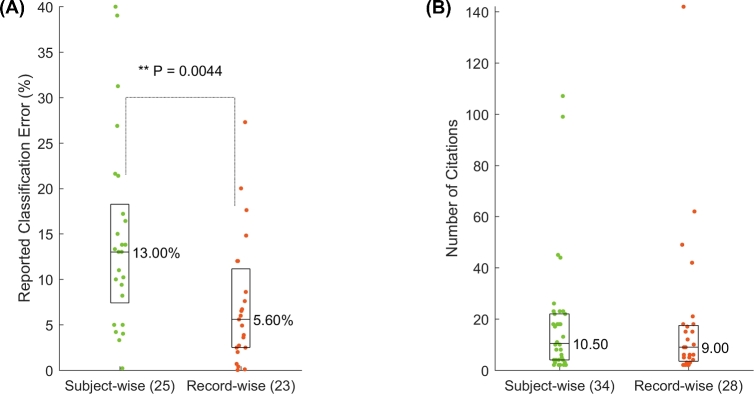
The difference between subject-wise and record-wise CV papers in (**A**) reported classification errors, and (**B**) number of citations. Each dot represents one paper, and boxes show the interquartile range. The horizontal lines inside the boxes indicate the medians.

Then, we extracted the reported accuracies from the papers included in our study. Due to lack of information, we could only extract accuracies from 47 of the 62 papers. As shown in Fig. 5A, for subject-wise CV papers, the median classification error (1 - accuracy) was 13.00%, more than twice that of record-wise CV papers, which was 5.60%. These values were significantly different (*P* < 0.01, two-tailed Wilcoxon rank sum test), exhibiting an inflated performance yielded by record-wise CV. Therefore, inappropriate CV method has led to dramatically more optimistic results.

However, the median number of citations received by papers in each category was very close (Fig. 5B), with subject-wise studies receiving 10.5 and record-wise 9.0 citations per paper. Therefore, whether or not a paper used the appropriate CV method did not affect the perceived impact of that paper in the field.

## Discussion

We evaluated the reliability of reported accuracies in studies that used machine learning and wearable sensor technology to predict clinical outcomes. Using a publicly available dataset and a simulation, we first showed that the record-wise CV method produces misleadingly high accuracy for global models. Then, we performed a systematic literature review and found that about 45% of the studies used record-wise CV for estimating the performance of their global models. As expected, the accuracies reported by the studies using the inappropriate method (record-wise) were higher than the ones estimated by the correct method (subject-wise). Therefore, it seems that a significant proportion of studies are dramatically overstating the ability of current machine learning algorithms to predict clinical outcomes.

Here we only considered one way that CV can be inappropriately used in clinical machine learning. In many prediction algorithms, in addition to the main parameters, there are hyperparameters that need to be adjusted, and very often, these are chosen such that the prediction error is minimized over the whole dataset. This makes the algorithms overfit the available datasets and thus not generalize to other datasets. The correct way for adjusting these hyperparameters is to further divide the training set into training and validation subsets, and then minimize the prediction error on those validation subsets rather than on the whole dataset. This approach allows for a proper evaluation of the generalizability of the algorithm [17].

Furthermore, while we only used one dataset, one could use any clinical dataset to show how mixing the subjects between training and validation sets can artificially increase the prediction accuracies. Clinical records often include information about physical or physiological characteristics of an individual, such as waist size or blood type, which do not vary much over time. For such data, if we use record-wise CV, the algorithms will already know the clinical outcome for an individual with specific characteristics. On the other hand, subject-wise CV will ensure that there is no way for the algorithm to exploit such shortcuts. Therefore, choosing the appropriate CV method is particularly important in clinical prediction applications.

The use of machine learning for clinical predictions is growing in popularity [25], although significant challenges lie ahead. For example, small datasets or the presence of rare conditions might limit the ability of an algorithm to generalize. In such scenarios, complementary approaches such as active learning or interactive machine learning [26, 27] could be beneficial. Nevertheless, the continuous increase in the computing power of electronic devices is contributing to the growth in the application of machine learning to health informatics. We are at the point where current mobile devices are as powerful as supercomputers from a few decades ago. This means that we can run very complex algorithms, on enormous amounts of data, using relatively cheap devices in a short period of time. In addition, the emergence of more advanced measurement devices with high spatial and temporal resolution (e.g., [28, 29]) requires the use of techniques that can analyze such large amounts of high-dimensional data, and in those applications, machine learning is replacing traditional data analysis. As such, machine learning tools are now used by investigators who might not have the proper training, which can open the door to misuse.

Even the most advanced algorithms need to be evaluated using appropriate validation methods. Proper validation helps to estimate the true predictive power of machine learning algorithms, which is crucial for the progress in the field. Inappropriate validation procedures, on the other hand, can lead to unreliable results, which might contribute to the problem of irreproducibility of research findings [31, 32] and thereby undermine the trust in both medicine and data science. Only with meaningful validation can the transition into machine learning-driven, data-rich medicine succeed.

### Availability and requirements

Project name: Cross-validation Project

Project home page: https://github.com/sosata/CrossValidation

Operating system(s): Platform independent

Programming language: MATLAB

Other requirements: MATLAB 2015a or higher

License: MIT License

Any restrictions to use by non-academics: No

### Availability of supporting data and materials

The human activity recognition dataset “Smartphone-Based Recognition of Human Activities and Postural Transition Data Set” from the UC Irvine Machine Learning Repository is publicly available at https://archive.ics.uci.edu/ml/datasets/Human+Activity+Recognition+Using+Smartphones.

The code for generating the simulated dataset is available at the GitHub repository: https://github.com/sosata/CrossValidation.

The systematic review results are available as a supporting file review_results.xlsx. Results of the literature review, used to generate Fig. 5 of the paper, and Matlab data files, which contain classification errors used to generate Fig. 3, are hosted in the *GigaScience* GigaDB repository [33], together with an archival copy of the Github repository.

### Additional files

**Additional files:** review_results.xlsx

**Additional files:** Lonini_et_alPrepubHistory.pdf

### Abbreviations

CV: Cross-validation; PD: Parkinson's Disease

### Ethics approval and consent to participate

The human activity recognition dataset used in our study was collected by a group of researchers at the University of Genova, Italy, and Polytechnic University of Catalonia, Spain. The authors of this paper had no involvement in the data collection.

### Consent for publication

Not applicable.

### Competing interests

The authors declare that they have no competing interests.

### Funding

This study was supported by the following National Institute of Health grants: 5R01NS063399, P20MH090318, and R01MH100482. Authors AJ and LL were supported by CBrace 80795 Otto Bock Healthcare Products, GmBH. The funding bodies had no role in the design of the study and collection, analysis, and interpretation of data and in writing the manuscript.

### Author contributions

Design: SS, LL, and KK. Data analysis: SS, LL, and KK. Systematic review: SS and LL. Writing: SS, LL, AJ, DM, and KK.

### Editor's note added in proof

Perspectives on the implications of this article are further discussed in an accompanying review by Max A Little et al. [34].

## Supplementary Material

GIGA-D-16-00098_Original_Submission.pdf

GIGA-D-16-00098_Revision_1.pdf

Response_to_reviewer_comments_Original_Submission.pdf

Additional files: review_results.xlsx

Additional files: Lonini_et_alPrepubHistory.pdf

## Footnotes

1 In *k*-fold cross-validation (CV), the dataset is split into *k* subsets, called *folds*. The algorithm is trained on *k-1* folds and tested on the remaining (validation) fold. The process is repeated until the algorithm is tested on all folds, and usually the average accuracy across all test folds is reported as the overall accuracy of the algorithm [17].

2 By definition, sensitivity = TP/(TP+FN), specificity = TN/(TN+FP), and accuracy = (TP+TN)/(TP+FN+TP+TN), where TP, FN, TN, and FP stand for the number of true positive, false negative, true negative, and false positive incidents, respectively. Since in general min(*a*/*b, c*/*d*) ≤ (*a*+*b*)/(*c*+*d*) ≤ max(*a*/*b, c*/*d*), the accuracy must be bounded by sensitivity and specificity.
