# Supplementary material for: The need to approximate the use-case in clinical machine learning
Source: Gigascience. 2017 Mar 15;6(5):1–9. doi: 10.1093/gigascience/gix019 (PMC5441397; doi:10.1093/gigascience/gix019)
Supplement: GIGA-D-16-00098_Original_Submission.pdf [file gix019_GIGA-D-16-00098_Original_Submission.pdf]

Manuscript
Click here to download Manuscript Manuscript_clean.docx
Click here to view linked References

The need to approximate the use-case in clinical machine learning

Sohrab Saeb[a,b,*], Luca Lonini[b,c,*] , Arun Jayaraman[b,c], David C. Mohr[a], Konrad P. Kording[b]

[a] Department of Preventive Medicine, Northwestern University, Chicago, USA

[b] Department of Physical Medicine and Rehabilitation, Northwestern University, Chicago, USA

[c] Max Nader Lab for Rehabilitation Technologies and Outcomes Research, Rehabilitation Institute of Chicago, Chicago, USA

* The first and the second author contributed equally to this work.

Corresponding author: Luca Lonini, Rehabilitation Institute of Chicago, 345 E. Superior St, Suite 1682, Chicago, IL 60611. Email: luca.lonini@northwestern.edu

Abstract

Background. The availability of smartphone and wearable sensor technology is leading to a rapid accumulation of human subject data, and machine learning is emerging as a technique to map that data into clinical predictions. As machine learning algorithms are increasingly used to support clinical decision making, it is vital to reliably quantify their prediction accuracy. Cross-validation is the standard approach where the accuracy of such algorithms is evaluated on data the algorithm has not seen during training. However, for this procedure to be meaningful, the relationship between the training and validation set should mimic the relationship between the training set and the dataset expected for the clinical use. Here we compared two popular cross-validation methods: record-wise and subject-wise. The subject-wise procedure mirrors the clinically relevant use-case scenario of diagnosing/identifying patterns in newly recruited subjects. The record-wise strategy has no such interpretation.

Results. Using both a publicly available dataset and a simulation, we found that record-wise cross-validation often massively overestimates the prediction accuracy of the algorithms. We also conducted a systematic review of the relevant literature, and found that this erroneous method is used by almost half of the retrieved studies that used accelerometers, wearable sensors, or smartphones to predict clinical outcomes.

Conclusions. As we move towards an era of machine learning based diagnosis and treatment, using proper methods to evaluate their accuracy is crucial, as erroneous results can mislead both clinicians and data scientists.

Keywords: Machine learning, cross-validation, clinical outcomes, rehabilitation outcomes, prediction accuracy, diagnosis, smartphones, wearable technology.

Background

Machine learning has evolved as the branch of artificial intelligence that studies how to solve tasks by learning from examples rather than being explicitly programmed. Machine learning has grown massively over the past decades, with countless applications in technology, marketing, and science [1]. Almost every smartphone nowadays includes speech recognition. Social media and e-commerce websites filter contents and recommend products based on the user's interests, and scientific data, from astrophysics [2] to neurophysiology [3] and medicine [4], are analyzed using machine learning algorithms.

In medicine, a great hope for machine learning is to automatically detect or predict disease states as well as assist doctors in diagnosis, using data collected by phones and wearable sensors. A driving factor is that people carry these devices with them most of the time, and thus a growing amount of daily life data, such as physical activities [5,6], is becoming available. Indeed, an increasing number of studies apply machine learning to the data collected from these devices for clinical prediction purposes. Examples include detecting cardiovascular diseases [7], falls [8], measuring rehabilitation outcomes in stroke and amputees [9,11], monitoring Parkinson's disease symptoms [12-14], and detecting depression [15,16].

The majority of machine learning algorithms used for clinical predictions are based on the supervised learning approach, which can be summarized in the following steps: first, a set of features is computed from the raw sensor data. These features are typically engineered depending on the specific application; e.g., one feature could be the maximum heart rate in a given time interval. Features are then fed into a machine learning classifier, the parameters of which are adjusted to map each input data point (feature vector or *record*) to its corresponding label, e.g., "healthy". Once the classifier is trained on enough data, it can be used to perform predictions on new subjects using their features; e.g., do their features predict that they are healthy?

A crucial stage of this process is to assess the prediction accuracy of the trained machine learning algorithm. The standard approach is to use cross-validation (CV) [17], where the data is split into training and test subsets. Splitting data into training and test subsets can be done in various ways, such as leave-one-out, leave-*p*-out, *k*-fold, and Monte-Carlo [18]. The classifier is trained on the training set, while its accuracy measured on the test set. The aim of this process is to assess the ability of the machine learning algorithm to generalize to new data.

The goal of typical machine learning applications in medicine is to build a model that can generalize to new subjects, e.g. subjects who should be automatically diagnosed. The proper form of CV in clinical machine learning is thus *subject-wise*: here the training and test sets contain records from different subjects; therefore, the performance of the classifier is measured on a new subject whose data has *not* been used for training (Figure 1A). Nevertheless, many studies employ *record-wise* CV, which randomly splits data into training and test sets regardless of which subjects they belong to. Therefore, records from the same subject are present in both training and test sets (Figure 1B). In this way, the machine learning algorithm can find an association between unique features of a subject (e.g., walking speed) and their clinical state, which automatically improves its prediction accuracy on their test data. As a consequence, the record-wise CV method can significantly overestimate the predicted accuracy of the algorithm.

Figure 1. A visualization of subject-wise (A) and record-wise (B) cross-validation (CV). Data is split into training (blue) and test (yellow) sets to evaluate the performance of machine learning algorithms. Each box represents a record from each subject. While subject-wise CV only uses data from other subjects (green arrows), record-wise in addition uses data from the same test subject (red arrows) to predict its state. The problem is, that if the algorithm can (implicitly) detect the identity of the person based on the features, it can automatically also “diagnose” the disease.

As this statistical problem is central to our paper, we start explaining the problem with a hypothetical example. Imagine we recruit 4 subjects, 2 healthy and 2 affected by Parkinson’s disease (PD). We have a machine learning algorithm that estimates if a person has PD based on their walking speed. Let our two healthy subjects have constant walking speeds of 1 meter per second (m/s) and 0.4 m/s, and our two PD patients 0.6 m/s and 0.2 m/s. If we do subject-wise CV, we will be unable to predict the performance of the slow healthy subject as well as the fast PD subject, resulting in a prediction accuracy of roughly 50%. Now for record-wise CV, let us have 10 records for each subject. To predict the first of the 10 measurements of the fast healthy subject we would also use the other 9 measurements of that same subject. We would thus be able to certainly conclude that this subject is healthy. The same would be true for the slow healthy subject. After all, none of the PD patients has a walking speed of 0.4 m/s. Subject identification, which we know to be relatively easy, thus replaces disease recognition which we know to be hard. As such, record-wise CV would give us a 100% accuracy which is clearly not supported by the data, and therefore the algorithm will not generalize.

The aim of this paper is (1) to demonstrate the potential threat to the clinical prediction literature when the wrong CV methods are used, and (2) to examine how widespread the problem is. We examine the first aim by showing that record-wise and subject-wise CV yield dramatically different results, with record-wise CV massively overestimating the accuracy. We demonstrate this by using a publicly available dataset on human activity recognition, as well as a simulation that shows how subject-specific and disease-specific factors interact. For the second aim, we conduct a systematic literature review to quantify the prevalence of this problem in studies that use smartphone and wearable sensor data to predict clinical and rehabilitation outcomes. We report the proportion of papers using record-wise CV against those using subject-wise CV, along with their classification accuracies and number of citations.

Human Activity Recognition

Data Description and Methods

First, we evaluated how record-wise and subject-wise cross-validation (CV) would be different in a real dataset. We chose a publicly available human activity recognition dataset [19,20] which contained recordings of 30 subjects performing 6 activities: sitting, standing, walking, stair climbing up/down, and laying down. Data consisted of recordings from the accelerometer and gyroscope sensors of a smartphone carried by the subjects. Each data record was a vector of 561 features computed from the sensors signal over a time window of 2.56 s. The dataset contained a total of 10299 records, with an average of 343 records per subject and an approximately equal number of records per activity.

We used MATLAB R2015a for the analysis. We used random forests [21] for classification. A random forest is an ensemble of decision trees, with each tree providing a prediction about the class of the input data. The forest's prediction is determined by averaging over the predictions of individual trees. Each tree in a random forest only sees a subset of features and a subset of input data samples. A random forest, thus, has fewer parameters to tune, which makes it less prone to overfitting and a better candidate for generalization to unseen data. We also found random forests to perform well in our previous activity recognition study [22]. Therefore, random forests were an appropriate choice for this study.

For each of the record-wise and subject-wise methods, we used 2, 10, or 30 subjects, and *k*-fold CV [a] with *k* = 2, 10, or 30. For record-wise, data was randomly split into CV folds regardless of which subject it came from. For subject-wise, we split data by subjects such that training and test folds contained records from different subjects. In both methods, the classifier was trained on all but one fold and tested on the remaining fold. The number of trees for the random forest classifier was set to 50, which

was optimized based on the out of bag error [21]. We repeated the training procedure 100 times, such that new subjects and folds were randomly generated in each repetition.

Analyses

We started by evaluating the performance of subject-wise CV on the activity recognition dataset. When using 2 folds and 2 subjects only, the error rate started at a value of 27% and, as the number of subjects increased to 30, it decreased significantly and reached 9% (Figure 2). Similarly, as the number of folds increased, i.e., data from more subjects was used for training the classifier, the error rate decreased and leveled around 7% with 30 folds (Figure 2, green lines).

We then trained the classifier using record-wise CV and used the same procedure to assess how the error changed as a function of number of subjects and folds. Interestingly, the classification error already started at a value of 2% when using data from 2 subjects, and did not significantly change when either number of subjects or folds increased (Figure 2, orange lines). Therefore, regardless of the amount of data used for training the classifier, record-wise CV significantly overestimated the classification accuracy on this dataset.

Figure 2. Effect of Subject-wise and record-wise CV on the classification error for the UCI activity recognition dataset. As the number of folds (x-axis) used to perform CV increases, the error tends to decrease. Similarly, performance improves when the number of subjects increases (symbols denote total number of subjects used to train and test the classifier). Record-wise CV significantly inflates the predicted accuracy (orange) as compared to subject-wise CV (green). Error bars indicate 95% confidence intervals.

Simulated Clinical Prediction

Data Description and Methods

In the second part of our study, we generated a simulated dataset to find out which properties of human subject data make the performance of subject-wise and record-wise CV methods different. Specifically, we were interested in two properties: *cross-subject variability*, and *within-subject variability*. Cross-subject variability is the variability in data that is observed across the subjects. In a diagnosis scenario, this property captures the effect of the disease, or the features that distinguish healthy subjects from sick. Within-subject variability represents the variability observed when multiple samples are recorded from a single subject. In clinical data, this property is usually related to the changes in the disease trajectory, as well as changes in the physiology of the subjects.

We used a generative model to create the simulated dataset. This model combined cross-subject and within-subject variabilities to generate an observation as the following:

$$y_{s,r,n} = a\beta_s + bu_{s,n} + cv_{s,r,n} + d\varepsilon_n \qquad (1)$$

where $y_{s,r,n}$ is the observed value for feature *n* ($n \in \{1,2,\dots,N\}$) in record *r* ($r \in \{1,2,\dots,R\}$) collected from subject *s* ($s \in \{1,2,\dots,S\}$). *N*, *R*, and *S* are the number of features, the number of records per subject, and the total number of subjects, respectively. β_s encodes disease effects, or fixed effects, with 1 for patients and -1 for healthy subjects. $u_{s,n} \sim \mathcal{N}(0,1)$ accounts for the random cross-subject variability, or random effects, and $v_{s,n,r} \sim \mathcal{N}(0,1)$ represents the within-subject variability. Finally, $\varepsilon_n \sim \mathcal{N}(0,1)$ is the population-level feature-generating process. *a, b, c*, and *d* are constant parameters, where *b* and *c* control the proportions of cross- and within-subject variability, respectively.

We generated datasets with variable number of subjects, from 4 to 32. These numbers were within the range used in the reviewed studies, as well as in the activity recognition dataset. In each dataset, half of the subjects were set to diseased ($\beta_s = 1$) and half were healthy ($\beta_s = -1$). We used *N* = 10 features and *R* = 100 records for each subject. We set *a* = 0.5, accounting for a disease effect size of (1-(-1)) x 0.5

= 1, and *d* = 0.1. We varied *b* and *c* between 0 to 2 with increments of 0.1 to test the effect of cross- and within- subject variability on the cross-validation results.

Similar to the activity recognition dataset, we trained and tested random forests on the simulated dataset, using both record-wise and subject-wise methods, to predict whether a record came from a patient ($\beta_s = 1$) or a healthy subject ($\beta_s = -1$). For record-wise, we randomly split the dataset into 50% training and 50% test, regardless of the subject IDs. For subject-wise, we did the same but we ensured that same subjects were not present in both training and test sets. For each CV method and each value of *b* and *c*, we measured the classification error on the test set. The simulation was repeated 10 times for each number of subjects and each value of *b* and *c*, and the average prediction error across the repetitions was calculated. The generative model code is available for download as detailed in the section "Availability of Supporting Data and Materials".

Finally, we wanted to see how the prediction errors evaluated by subject-wise and record-wise CV methods compared to the true prediction error. To find the true prediction error, we generated an additional dataset of 10 subjects, which were not used for cross-validation, using the same generative model (Equation 1). We calculated the true prediction error by evaluating the performance of each of the trained classifiers on this new dataset. In this way, in addition to evaluating the difference between subject-wise and record-wise prediction errors, we could see which of them was closer to the true prediction error.

Analyses

We quantified how cross-subject variability (*b*) and within-subject variability (*c*) contributed to the classification errors in either of the two CV methods (see Equation 1). As in the activity recognition task, we used a random forest classifier. Figure 3A shows how classification error changed with *b* and *c*, for 4,

12, and 32 subjects. In all scenarios, for small values of *b* and *c* the classification error was small. This is because when both within- and cross-subject variabilities are low, the disease effect is more prominent in the features, helping the classifier to distinguish between healthy and diseased subjects. For higher values of *c*, classification errors were higher, especially for the subject-wise method.

Figure 3. Classification error in a simulated dataset for record-wise and subject-wise cross-validation (CV) methods, as a function of cross-subject (*b*) and within-subject (*c*) variability (A), and number of subjects (B). The number of features is set to 10. (A) Each column shows the classification error for both CV methods as a function of *b* and *c* for a given number of subjects (4, 12, and 32). Brighter colors indicate higher classification error values (black = 0; white = 1). (B) The mean and the standard deviation of classification error, across the values of *b* and *c*, for subject-wise and record-wise methods, as a function of number of subjects.

Increasing the cross-subject variability (*b*) alone did not increase the classification error for the record-wise method, when the number of subjects was small (top left panel). Indeed, in the record-wise CV, the classifier was already informed about *b* by having samples from all or most of the subjects in its training set. For the subject-wise method, on the other hand, increasing *b* dramatically increased the classification error (bottom left panel). Nevertheless, when more subjects were used, the classification error increased in both CV methods, but remained lower for the record-wise method (top right versus bottom right panel).

Overall, as shown in Figure 3B, record-wise CV underestimated the classification error relative to the subject-wise CV method. This was further confirmed by comparing the prediction error yielded by each CV method with the true error, shown by dashed lines in Figure 3B, which was computed by testing each trained model on a new dataset that was not used for CV. The prediction error calculated using the subject-wise CV (black line) method was closer to the true error, while the record-wise method (red line)

was massively underestimating it. In addition, subject-wise CV errors closely followed the true classification error as the number of training subjects changed.

The difference between the record-wise and subject-wise error was largest when the number of subjects was small, and it gradually decreased as we increased the number of subjects. This is because as we increase the number of subjects, it becomes harder for the record-wise classifier to identify subjects based on their data, thereby losing its advantage over the subject-wise method. Nevertheless, even for relatively large number of subjects, record-wise CV leads to an underestimated classification error, while the subject-wise CV has a significantly closer estimate of the true performance of the algorithm

Systematic Review

In the last part of our study, we systematically reviewed the literature to find out what percentage of the published studies used record-wise versus subject-wise cross-validation (CV). We specifically looked for the studies which used both machine learning and smartphone or wearable sensor technology for clinical predictions. This process had three main steps: (1) searching for relevant papers; (2) excluding papers which did not meet our eligibility criteria; and (3) determining the CV type used in each paper. This process was consistent with PRISMA guidelines (Figure 4). The papers were reviewed and analyzed by two authors (SS, LL). A total of 4 discrepancies were resolved by consensus.

Figure 4. PRISMA flowchart.

Search Strategy

We wanted to find the studies which contained at least one of the following keywords in their title: “wearable”, “smartphone”, or “accelerometer”. In addition, to account for the clinical applicability, the papers had to have one of the following keywords in their text: “diagnosis”, “disease”, or “rehabilitation”. Finally, the papers had to contain “cross-validation” at least once, and be published after 2010. For this purpose, we used Google Scholar [23] with the following search query:

(intitle:wearable OR intitle:smartphone OR intitle:smartphones OR intitle:accelerometer) AND (diagnosis OR disease OR rehabilitation) "Cross-Validation" (from 2010)

Eligibility Criteria

1) Peer-reviewed studies published in English.

2) Studies which used a machine learning technique to predict a clinical or rehabilitation outcome.

3) Studies which used cross-validation (CV) to assess the performance of their algorithms.

4) Studies which used more than one data point (record) for each subject. In fact, when there is only one record per subject, record-wise CV and subject-wise CV are the same.

5) Studies which did not use *personal* models, which are trained and tested on each individual subject separately.

6) Studies which were not review of the literature or book chapter.

7) Studies for which the CV type was not “unknown” (see Determining the Cross-validation Type).

Determining the Cross-validation Type

After finding the papers which met our eligibility criteria, we grouped them into the ones which used record-wise CV and the ones that used subject-wise CV. We assigned a paper to the subject-wise group if one or more of the following conditions were satisfied:

1) The authors used the term “subject-wise” or “leave-one-subject-out” when explaining their cross-validation strategy.
2) The authors mentioned that they tested their algorithms on subjects that were not included in the training set.
3) We did not find any overlap between the subject IDs in training and test datasets, where subject IDs were provided.

We assigned a paper to the record-wise group if one or more of the following conditions were satisfied:

1) The number of cross-validation folds was greater than the number of subjects.
2) The authors mentioned that they randomly split the whole dataset into training and test subsets.
3) We found an overlap between subject IDs between the training and test datasets, where subject IDs were provided.

If none of the 6 conditions above were satisfied, we labeled the CV type as “unknown”.

Extracting Other Metrics

We also wanted to see if subject-wise and record-wise CV studies were different in the number of citations they received, as well as in their reported classification accuracy. For the number of citations, we used the information provided by Google Scholar. For the accuracy, however, we analyzed the full-text papers. Since papers used different metrics to report the classification accuracy of their algorithms, we used the following rules to find their accuracies:

1) Where a single accuracy or classification error value was reported, we directly used that.

2) Where multiple accuracies were reported for different conditions or classes, we used the average accuracy.

3) Where accuracies were reported for different types of classifiers or feature sets, we used the highest accuracy.

4) Where sensitivity and specificity were reported and their difference was less than 2%, we used their average as accuracy. This is supported by the fact that accuracy is bounded by sensitivity and specificity [b].

5) Where F1-score was reported, or precision and recall were reported from which we could calculate the F1-score, we used it instead of the accuracy.

6) We did not extract the classification accuracy from the papers which only reported root-mean-square error (RMSE), normalized RMSE (NRMSE) or similar metrics.

Analyses

A total of 369 papers were initially retrieved from Google Scholar. Of these, we screened the first 200 papers. 113 papers were excluded upon the review of the title and the abstract because they: (1) reported studies that were not related to any clinical or rehabilitation outcomes (n = 98), (2) were review of the literature or book chapters (n = 10), (3) were dissertation reports (n = 4), or (4) used personal models (n = 1). We retrieved the full text of the remaining 87 papers. Out of these, we excluded 25 more papers, based on the criteria detailed in Figure 4. We included the remaining 62 papers in our systematic review. We determined which of these papers used the subject-wise or record-wise cross-validation method, and found that 28 used record-wise while 34 used subject-wise CV. Therefore, about 45% of the reviewed papers used the wrong (record-wise) CV method.

Then, we extracted the reported accuracies from the papers included in our study. Due to lack of information, we could only extract accuracies from 47 out of the 62 papers. As shown in Figure 5A, for

subject-wise CV papers, the median classification error (1 - accuracy) was 13.00%, more than twice that of record-wise CV, which was 5.60%. These values were significantly different (P<0.01, two-tailed Wilcoxon rank sum test), exhibiting an inflated performance yielded by record-wise CV. Therefore, improper cross-validation has led to dramatically “better” results.

However, the median number of citations received by papers in each category were very close (Figure 5B), with subject-wise studies receiving 10.5 and record-wise 9.0 citations per paper. Therefore, whether a paper used the correct or wrong CV method did not affect the perceived impact of that paper in the field.

Figure 5. The difference between subject-wise and record-wise cross-validation papers in (A) reported classification errors, and (B) number of citations. Each dot represents one paper, and boxes show the interquartile range. The horizontal lines inside the boxes indicate the medians.

Discussion

We evaluated the reliability of reported accuracies in studies that used machine learning and wearable sensor technology to predict clinical outcomes. Using a publicly available dataset and a simulation, we first showed that the record-wise cross-validation (CV) method produces misleadingly high accuracy. Then, we performed a systematic literature review and found that about 45% of the studies used record-wise CV. As expected, the accuracies reported by the studies using the wrong method (record-wise) were higher than the ones reported by correct (subject-wise) papers. Therefore, it seems that a significant proportion of studies are dramatically overstating the ability of current machine learning algorithms to predict clinical outcomes.

Here we only considered one way that cross-validation can go wrong. In fact, the problem is not limited to using the record-wise method. In many prediction algorithms, there are hyperparameters that need to be adjusted, and very often, these are chosen such that the prediction error is minimized. This makes the algorithms overfit the data and thus not generalize to other datasets. The correct way for learning these hyperparameters is to further divide the training set into training and validation subsets, and then minimize the prediction error on those validation subsets rather than on the whole dataset. This approach allows for a proper evaluation of the generalizability of the algorithm [17].

Furthermore, while we only used one dataset, one could use any clinical dataset to show how mixing the subjects between training and validation sets can artificially increase the prediction accuracies. Clinical records often include information about physical or physiological characteristics of an individual, such as waist size or blood type, which do not vary much over time. For such data, if we use record-wise CV, the algorithms will already know the clinical outcome for an individual with specific characteristics. On the other hand, subject-wise CV will ensure that there is no way for the algorithm to exploit such shortcuts. Therefore, choosing the right CV method is particularly important in clinical prediction applications.

The use of machine learning for clinical predictions is growing in popularity [24], although significant challenges lie ahead. For example, small datasets or the presence of rare conditions might limit the ability of an algorithm to generalize. In such scenarios, complementary approaches such as active learning or interactive machine learning [25,26] could be beneficial. Nevertheless, the continuous increase in the computing power of electronic devices is contributing to the application of machine learning to health informatics. We are at the point where current mobile devices are as powerful as supercomputers from a few decades ago. This means that we can run very complex algorithms, on enormous amounts of data, using relatively cheap devices in a short period of time. In addition, the emergence of more advanced measurement devices with high spatial and temporal resolution (e.g., [27,28]) requires the use of techniques that can analyze such large amounts of high-dimensional data,

and in those applications, machine learning is replacing traditional data analysis. As such, machine learning tools are now used by investigators who might not have the proper training, which can open the door to misuse.

The most advanced algorithms and the highest quality datasets, if used with wrong CV methods, lead to meaningless results. Such erroneously positive results threaten the progress in the field. A direct consequence is the waste of resources on the projects that are based on fallacious findings. Furthermore, such results might contribute to the problem of irreproducibility of research findings [29,30] and thereby undermine the trust in both medicine and data science. Only with meaningful validation procedures can the transition into machine learning driven, data-rich medicine succeed.

Availability and requirements

Project name: Cross-validation Project

Project home page: https://github.com/sosata/CrossValidation

Operating system(s): Platform independent

Programming language: MATLAB

Other requirements: MATLAB 2015a or higher

License: MIT License

Any restrictions to use by non-academics: No

Availability of supporting data and materials

The human activity recognition dataset “Smartphone-Based Recognition of Human Activities and Postural Transition Data Set” from the UC Irvine Machine Learning Repository is publicly available at https://archive.ics.uci.edu/ml/datasets/Human+Activity+Recognition+Using+Smartphones

The code for generating the simulated dataset is available at the GitHub repository: https://github.com/sosata/CrossValidation

The systematic review results are available as a supporting file review_results.xlsx.

Declarations

List of abbreviations

CV: Cross-validation

PD: Parkinson’s Disease

Ethics approval and consent to participate

The human activity recognition dataset used in our study was collected by a group of researchers at the University of Genova, Italy, and Polytechnic University of Catalonia, Spain. The authors of this paper had no involvement in the data collection.

Consent for publication

Not applicable.

Competing interests

The authors declare that they have no competing interests.

Funding

This study was supported by the following National Institute of Health grants: 5R01NS063399, P20MH090318, and R01MH100482. Authors AJ and LL were supported by CBrace 80795 Otto Bock

Healthcare Products, GmBH. The funding bodies had no role in the design of the study and collection, analysis, and interpretation of data and in writing the manuscript.

Authors' contributions

Design: SS, LL, KK

Data Analysis: SS, LL, KK

Systematic Review: SS, LL

Writing: SS, LL, AJ, DM, KK

Acknowledgements

The authors thank Joshua Glasier for helpful comments on the manuscript, and the creators of the UCI Machine Learning Repository for making the human activity recognition dataset available to researchers.

Endnotes

a. In *k*-fold cross-validation (CV), the dataset is split into *k* subsets, called *folds*. The algorithm is trained on *k-1* folds and tested on the remaining (validation) fold. The process is repeated until the algorithm is tested on all folds, and usually the average accuracy across all test folds is reported as the overall accuracy of the algorithm [17].

b. By definition, sensitivity = TP/(TP+FN), specificity = TN/(TN+FP), and accuracy = (TP+TN)/(TP+FN+TP+TN), where TP, FN, TN, and FP stand for the number of true positive, false negative, true negative, and false positive incidents, respectively. Since in general $\min(a/b\ ,\ c/d) \leq (a+b)/(c+d) \leq \max(a/b\ ,\ c/d)$, the accuracy must be bounded by sensitivity and specificity.

References

1. Jordan MI, Mitchell TM. Machine learning: Trends, perspectives, and prospects. Science. 2015 Jul 17;349(6245):255-60. DOI: 10.1126/science.aaa8415
2. Dieleman S, Willett KW, Dambre J. Rotation-invariant convolutional neural networks for galaxy morphology prediction. Monthly Notices of the Royal Astronomical Society. 2015 Jun 21;450(2):1441-59. DOI: 10.1093/mnras/stv632
3. Jonas E, Kording K. Automatic discovery of cell types and microcircuitry from neural connectomics. eLife. 2015 Apr 30;4:e04250. DOI: http://dx.doi.org/10.7554/eLife.04250
4. Donsa, K., Spat, S., Beck, P., Pieber, T. R. & Holzinger, A. 2015. Towards personalization of diabetes therapy using computerized decision support and machine learning: some open problems and challenges. In: Holzinger, A., Roecker, C. & Ziefle, M. (eds.) Smart Health, Lecture Notes in Computer Science LNCS 8700. Heidelberg, Berlin: Springer, pp. 235-260, doi:10.1007/978-3-319-16226-3_10.
5. Dobkin BH. Wearable motion sensors to continuously measure real-world physical activities. Current opinion in neurology. 2013 Dec;26(6):602. DOI: 10.1109/iembs.2008.4650384
6. Piwek L, Ellis DA, Andrews S, Joinson A. The rise of consumer health wearables: promises and barriers. PLoS Medicine. 2015 Dec 22. DOI: 10.1371/journal.pmed.1001953
7. Oresko JJ, Jin Z, Cheng J, Huang S, Sun Y, Duschl H, Cheng AC. A wearable smartphone-based platform for real-time cardiovascular disease detection via electrocardiogram processing. Information Technology in Biomedicine, IEEE Transactions on. 2010 May;14(3):734-40. DOI: 10.1109/titb.2010.2047865
8. Albert MV, Kording K, Herrmann M, Jayaraman A. Fall classification by machine learning using mobile phones. PloS one. 2012 May 7;7(5):e36556. DOI: 10.1371/journal.pone.0036556
9. Patel S, Hughes R, Hester T, Stein J, Akay M, Dy JG, Bonato P. A novel approach to monitor rehabilitation outcomes in stroke survivors using wearable technology. Proceedings of the IEEE. 2010 Mar;98(3):450-61. DOI: 10.1109/jproc.2009.2038727
10. Dobkin BH, Xu X, Batalin M, Thomas S, Kaiser W. Reliability and validity of bilateral ankle accelerometer algorithms for activity recognition and walking speed after stroke. Stroke. 2011 Aug 1;42(8):2246-50. DOI: 10.1161/strokeaha.110.611095

11. Albert MV, Deeny S, McCarthy C, Valentin J, Jayaraman A. Monitoring daily function in persons with transfemoral amputations using a commercial activity monitor: A feasibility study. PM&R. 2014 Dec 31;6(12):1120-7. DOI: 10.1016/j.pmrj.2014.06.006

12. Patel S, Lorincz K, Hughes R, Huggins N, Growdon J, Standaert D, Akay M, Dy J, Welsh M, Bonato P. Monitoring motor fluctuations in patients with Parkinson's disease using wearable sensors. Information Technology in Biomedicine, IEEE Transactions on. 2009 Nov;13(6):864-73. DOI: 10.1109/titb.2009.2033471

13. Bächlin M, Plotnik M, Roggen D, Maidan I, Hausdorff JM, Giladi N, Tröster G. Wearable assistant for Parkinson's disease patients with the freezing of gait symptom. Information Technology in Biomedicine, IEEE Transactions on. 2010 Mar;14(2):436-46. DOI: 10.1109/titb.2009.2036165

14. Arora S, Venkataraman V, Zhan A, Donohue S, Biglan KM, Dorsey ER, Little MA. Detecting and monitoring the symptoms of Parkinson's disease using smartphones: a pilot study. Parkinsonism & related disorders. 2015 Jun 30;21(6):650-3. DOI: 10.1016/j.parkreldis.2015.02.026

15. Canzian L, Musolesi M. Trajectories of depression: unobtrusive monitoring of depressive states by means of smartphone mobility traces analysis. In Proceedings of the 2015 ACM International Joint Conference on Pervasive and Ubiquitous Computing 2015 Sep 7 (pp. 1293-1304). ACM. DOI: 10.1145/2750858.2805845

16. Saeb S, Zhang M, Karr CJ, Schueller SM, Corden ME, Kording KP, Mohr DC. Mobile phone sensor correlates of depressive symptom severity in daily-life behavior: an exploratory study. Journal of medical Internet research. 2015 Jul;17(7). DOI: 10.2196/jmir.4273

17. Friedman J, Hastie T, Tibshirani R. The elements of statistical learning. Springer, Berlin: Springer series in statistics; 2001. DOI: 10.1007/b94608

18. Arlot, S., & Celisse, A. (2010). A survey of cross-validation procedures for model selection. Statistics surveys, 4, 40-79.

19. Lichman M (2013). UCI Machine Learning Repository – "Smartphone-Based Recognition of Human Activities and Postural Transitions Data Set" [https://archive.ics.uci.edu/ml/datasets/Smartphone-Based+Recognition+of+Human+Activities+and+Postural+Transitions]. Irvine, CA: University of California, School of Information and Computer Science.

20. Reyes-Ortiz, J. L., Oneto, L., Samà, A., Parra, X., & Anguita, D. (2016). Transition-aware human activity recognition using smartphones. *Neurocomputing*, *171*, 754-767. DOI: 10.1016/j.neucom.2015.07.085

21. Breiman L. Random forests. Machine learning. 2001 Oct 1;45(1):5-32. DOI: 10.1023/A:1010933404324

22. Saeb S, Körding K, Mohr DC. Making Activity Recognition Robust against Deceptive Behavior. PloS one. 2015 Dec 11;10(12):e0144795. DOI: 10.1371/journal.pone.0144795

23. Google Scholar. Google. 2016. https://scholar.google.com.

24. Sandstrom GM, Lathia N, Mascolo C, Rentfrow PJ. “Opportunities for smartphones in clinical care: the future of mobile mood monitoring.” J Clin Psychiatry. 2016 Feb;77(2):e135-7. DOI: 10.4088/jcp.15com10054

25. Holzinger, A. 2016. Interactive Machine Learning for Health Informatics: When do we need the human-in-the-loop? Springer Brain Informatics, 3, (2), 119-131, doi:10.1007/s40708-016-0042-6

26. Holzinger, A., Plass, M., Holzinger, K., Crisan, G., Pintea, C. & Palade, V. 2016. Towards interactive Machine Learning (iML): Applying Ant Colony Algorithms to solve the Traveling Salesman Problem with the Human-in-the-Loop approach. Springer Lecture Notes in Computer Science LNCS 9817. Heidelberg, Berlin, New York: Springer, doi:10.1007/978-3-319-45507-56.

27. Müller, Jan, et al. "High-resolution CMOS MEA platform to study neurons at subcellular, cellular, and network levels." *Lab on a Chip* 15.13 (2015): 2767-2780. DOI: 10.1039/c5lc00133a

28. Chittaranjan, Gokul, Jan Blom, and Daniel Gatica-Perez. "Mining large-scale smartphone data for personality studies." *Personal and Ubiquitous Computing* 17.3 (2013): 433-450. DOI: 10.1007/s00779-011-0490-1

29. Ioannidis JP. Why most published research findings are false. PLoS Med. 2005 Aug 30;2(8):e124. DOI: 10.1371/journal.pmed.0020124

30. Open Science Collaboration. Estimating the reproducibility of psychological science. Science. 2015 Aug 28;349(6251):aac4716. DOI: 10.1126/science.aac4716

Click here to download Manuscript Response to Reviewer 1.docx
Click here to view linked References

Response to Reviewer 1

First, we want to thank the reviewers for participating in this non-anonymous review process. Their responses have made our paper dramatically stronger. However, there are also a range of issues that we cannot agree with as we will detail below.

Records are not *i.i.d.*

The core of Dr. Little's argument is that we want our training and test subsets be identically-distributed in order to have maximally similar distributions, and since subjects have different distribution of covariates, we should sample from all subjects in training and test subsets to get similar distributions:

> Now, returning to the context that the authors investigate in this manuscript where there are small numbers of participants and the studies are of behavioural and/or biological phenomena, the chance that the data is drawn i.i.d. from the population distribution, and hence statistically homogenous, is small. More realistically, and from my own experience, the data is highly heterogenous. For example, we often find examples of individuals whose within-subject variability is the same as the between-subject variability across the entire group. Furthermore, the data is often *clustered* by each individual, that is, we will find that variables tend to group in value within an individual (for a real-world example of this, see Figure 2.1). With such clustering, it is obvious that we cannot split the data by individual and expect that the variables in the training and testing subsets have the same distribution (see Figure 2.2 for an illustration on synthetic data). In this situation, subject-wise CV is not statistically valid because the primary i.i.d. assumption is violated.

There clearly is variability across subjects – that is the point of the entire paper. But we cannot at all agree with the statistical conclusions of Dr. Little. We see a great deal of statistical confusion in this argument and we want to step through the logic very clearly.

The definition of a good statistical approach is that it estimates the relevant variables. In the context of machine learning in medicine, by definition, the relevant variable, the variable that doctors want to know about, is if we would implement the ML approach and then recruit more patients, how well would it do on these patients. That is what the real world use and that is what investors, doctors and other scientists want to use when evaluating how well a system works. So, let us formulate that very clearly and then come back to the *i.i.d.* issue. We want to estimate how well our machine learning system would work on a new batch of patients. Good performance means having an estimator that is minimally biased and has low variance. In other words, if we read an ML paper that shows great performance, the reader wants to take away that it will work in the real life scenario, which means it will work in a situation with new patients.

Now, let us get back to Dr. Little's comments. There are two potential *i.i.d.*s involved here, and his argument is confusing the two of them. First, when proper recruitment procedures are used, then subjects are in good approximation drawn *i.i.d.* from the overall population of subjects. In human subject studies, this means that we need to have a large and representative sample of the population.

However, in such a scenario, records are definitely non-*i.i.d.* In fact, as Dr. Little points out, records from the same subjects tend to be highly correlated to one another and are thus not independent. The point that we will predict worse by excluding records from the same subject is, in this context, a truism. But the fact that different subjects are distinct is the central problem. Also, the fact that records are not *i.i.d.*, even if subjects are, is an important fact of life. This is exactly the effect that makes record-wise cross-validation underestimate the errors as we will show clearer in the revised version of the paper.

The violation of *i.i.d.* across records is exactly what produces the erroneously low error rates of the record-wise strategy. Some of the variance, as Dr. Little rightfully points out, relates to the subject. The records from the same subject are not independent. They are correlated because they come from the same subject. And hence, whatever label or variable is estimated from the samples will effectively, improperly utilize subject-specific features to predict the disease. For a formal justification of the optimality of leave-subject-out cross-validation we want to refer the interested reader to Xu and Huang (2012). So to be clear, the leave-one-subject-out cross-validation has been proven to be correct. The record-wise cross-validation in this context violates the independence assumption, and without independence, it overestimates the prediction accuracy (and the new figure 3b very clearly shows this).

A detailed comparison with the ground truth

The goal of machine learning is to develop algorithms that not only work for the specific dataset they have been trained or cross-validated on, but to generalize to new data. But what does new data mean in clinical prediction problems? It depends on what the algorithm is developed for. If the algorithm is developed specifically for one subject, new data means new records from the same subject. This falls into the category of *personal* models which were excluded from our study. However, if the goal is diagnosis, meaning that we want to develop *global* algorithms that can predict a clinical outcome based on the records from a new subject, new data means new subjects. <u>Therefore, the true prediction error can be defined as the prediction error of the algorithm on new subjects from the same population, which have not been used for cross-validation. The goal of cross-validation is to estimate this error.</u>

In our simulation study in the paper (Simulated Clinical Prediction), we can estimate the true prediction error by generating an additional dataset, that is generated using the same generative model. The prediction error of the models that are trained and cross-validated on the main dataset is evaluated on this new dataset and will serve as the true prediction error. Then, we can compare the prediction errors estimated by cross-validation on the main dataset to the true error.

Here, we generated new data for 10 subjects and considered that as the new dataset. We evaluated each of the trained models in Figure 3B on this dataset. As the results show, the prediction error calculated using subject-wise cross-validation is a much better estimator of the true prediction error, and the record-wise method is massively underestimating it. Above all, besides obvious minor biases, we can see how subject-wise CV correctly tracks the variables of interest while record-wise CV does not.

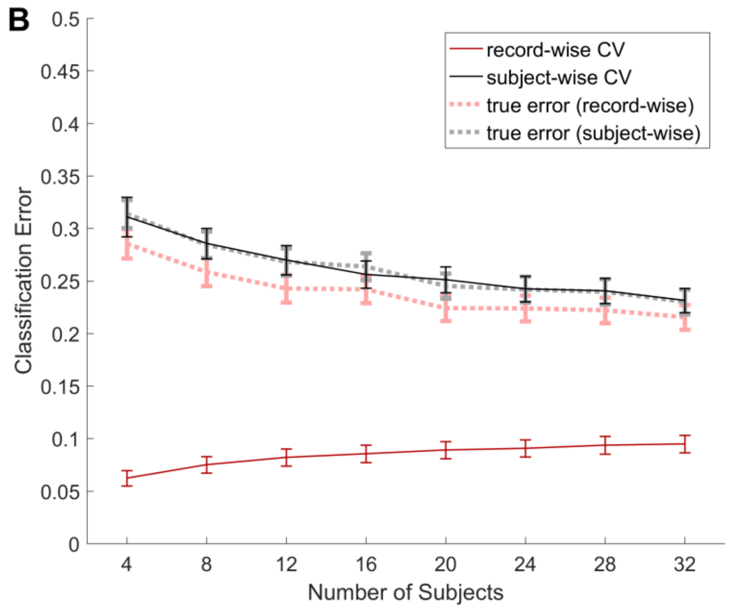

Figure 1. (Figure 3B in the new manuscript). Classification error on a simulated dataset for record-wise and subject-wise cross-validation (CV) methods, along with the true prediction errors computed on a new dataset, as a function of number of subjects. The number of features is set to 10. The mean and the 95% confidence interval of the classification error is shown.

The provided simulations (Figure 2.3) were grossly misleading

We reproduced the simulation that the reviewer has done in Figure 2.3 of the response letter. While we got the same results as the reviewer for record-wise RMSE results (Fig 2A), the results for subject-wise cross-validation depended on how many subjects were used for training and how many for test, since there were only 5 subjects in the dataset. We can only replicate the results in Figure 2.3 if we used 1 subject to train and the remaining 4 for test (Fig 2B). This, however, is an extremely unusual way to do subject-wise cross-validation. In fact, it makes no sense whatsoever, as generalizing from a single subject to a population is generally impossible. When we have such a small dataset, the common way to do subject-wise cross-validation is leave-one-out, also called jackknife. And in fact, if we cross-validate using the leave-one-subject-out method, the resulting RMSE looks like Fig 2C, which is very different from Fig 2B and what the reviewer has shown in his response, and correctly suggests that the quadratic model is the best fit. In other words, if done correctly then subject-wise-cross-validation will work just fine even for the tiny population chosen by the reviewer. We agree, however, that having too few subjects makes subject-wise CV impossible – and indeed, we do not feel ML papers can tell much about their population performance based on a very small sample.

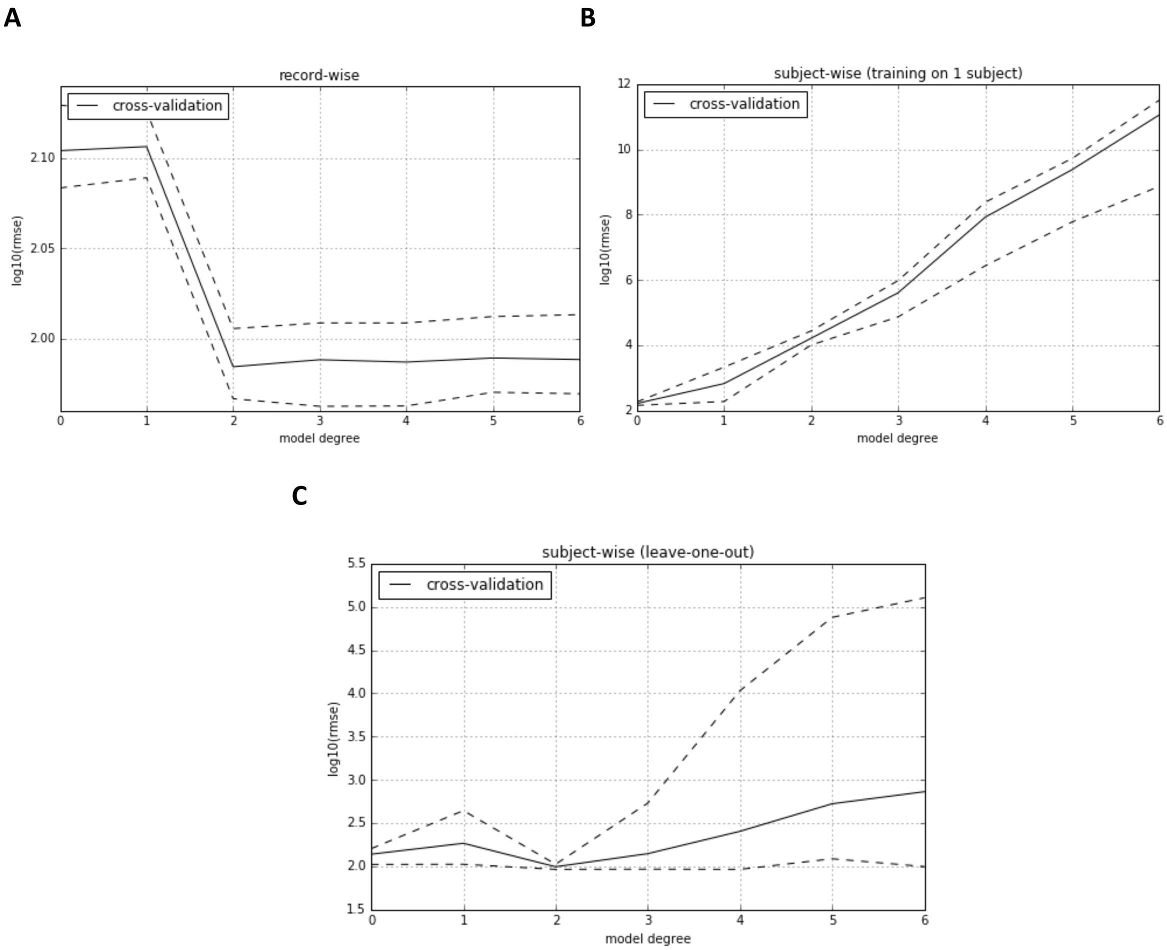

Figure 2. Reproduction of Figure 2.3 simulation in the reviewer's letter. The record-wise model's RMSE (A) shows that the quadratic model is probably the best fit, while the model trained on one subject and tested on the remaining 4 (B) suggests the constant model as the best fit, which is wrong. However, if leave-one-subject-out CV is used (C), the results look different, and the best model suggested by cross-validation is again quadratic.

While these simulations may be enough to show that the results shown in Figure 2.3 of the reviewer's letter are misleading, we also wanted to know how the subject-wise and record-wise RMSEs compared to the true prediction errors here. To calculate the true prediction error, we generated an additional subject using the same generative model, shown by green dots in Fig 3A. Then, we evaluated the prediction error of both subject-wise and record-wise models on this new subject. As the results in Fig 3B and 3C show, both models underestimate the error. However, the record-wise model has a much larger underestimation, and it especially fails at predicting the error for more complex models. Prediction errors estimated using subject-wise CV, on the other hand, are much closer to the true prediction error and worsen as model complexity increases, the same way that the true prediction error does. Again, these biases are exactly as they should be predicted.

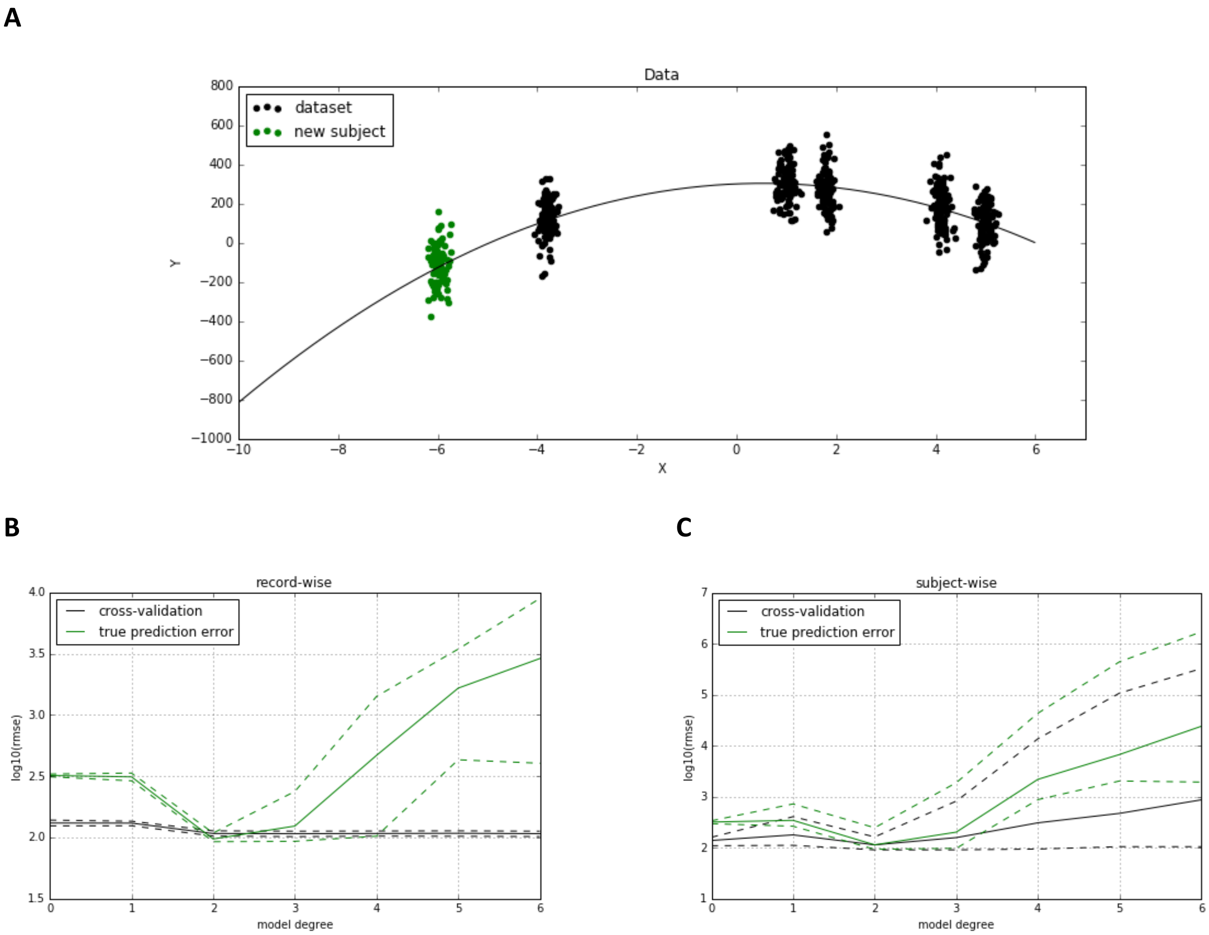

Figure 3. The goal of cross-validation is to estimate the true prediction error, which in this context can be defined as the predicted RMSE on a new subject, shown by green dots (A). While the record-wise CV is massively underestimating this generalization error (B, green), the cross-validation error estimated by subject-wise CV is much closer to the generalization error (C), and it correctly follows its trend over the complexity of the model. Solid lines show the mean, and dashed lines show the minimum and maximum values.

Response to specific arguments and comments

Comment: "But a huge amount is already known and written about this (e.g. read Hand, 2006) and this paper does not get us further towards this goal because of the fundamental misapprehension about the mathematical foundations of CV. Nor does the excessive hyperbole (see specific comments below) and the overblown polemical rant (that belongs on the editorial opinion pages, or Twitter, and not in a scientific article)".

To our knowledge, no one has previously shown the difference between subject-wise and record-wise cross-validation procedures in the way we have shown in this paper. The paper cited above (Hand, 2006) does not discuss the problem of subject-wise versus record-wise cross-validation, therefore, we cannot agree with this comment. Quite specifically, we believe that this is the first paper about subject vs

record-wise cross-validation. We believe that it is the first literature analysis in this area, that it is the first paper providing detailed insights into the biases produced by this approach. While we also do not perceive our paper as an “overblown polemical rant”, we did tone down the wording in line with the suggestions of both reviewers.

Comment: Figure 2.2. “As can be seen, while the data is drawn i.i.d. from a mixture distribution, the non-stratified (recording-wise) scheme leads to indistinguishable distributions for training and test sets, whereas, in the stratified (subject-wise) case, the train/test distributions are radically different. Therefore, covariate stratification (i.e. splitting by subject ID) violates an essential assumption of cross-validation.

In the reviewer’s simulation (Fig. 2.2), there are only 3 subjects. But generalizing from one subject to the others is almost meaningless and no one should be surprised by the algorithm failing in such a scenario – and no journal should publish ML papers with N=3. This is exactly why we need as many subjects as possible in our dataset. Again, it is not important here how low the cross-validation error is. The importance is how well the algorithms perform on new data, which obviously, given the sample size of 3, must be very bad. This bad performance is truly predicted by a subject-wise CV scheme, as Dr. Little points out, while the use of record-wise CV here would be completely misleading.

Comment: What are the practical limitations of CV, and what are the alternatives? The issues can be subtle and quite complex [Kubota et al., 2016]…. In my opinion, another, similarly valuable direction, is to raise awareness of more appropriate techniques which can be used in the situation where distributional mismatch occurs, e.g. covariate shift [Sugiyama et al., 2007, Shimodaira, 2000], domain adaptation [Blitzer et al., 2008], methods for class imbalance, sample selection bias and dealing with data drift [Hand, 2006].

Domain adaptation is about adapting algorithms to a new dataset with differently-distributed features (covariates). The focus of this paper is on how to train and cross-validate algorithms properly on an existing dataset, with non-shifting covariates. Let us first solve this problem and then we can move on to problems beyond CV – which should be part of another paper.

Title. This doesn’t make any sense. What do the authors mean exactly by “voodoo machine learning” and “clinical predictions”? The title needs to be changed to actually describe the topic of the paper.

The title is a nod to a famous preprint by Ed Vul that deals with other statistical mistakes in the literature. Realizing that this link will be lost to much of our audience we decided to delete it.

P2, L36: “however, several cross-validation methods exist and only some of them are statistically meaningful.” What exactly is meant by this? Yes, there are many CV methods [Arlot and Celisse, 2010, Shimodaira, 2000, Sugiyama et al., 2007], but they are all, by definition “statistically meaningful”. So, this statement needs to be corrected.

We now very clearly define what it means for a statistical procedure to be meaningful for a real world problem at hand. We thus also clarified this sentence.

Simulated Clinical Prediction. This is an interesting experiment but it is not sufficiently well described to allow replication. Are the +1/-1 labels which indicate healthy/disease status generated at random? If so, what is their distribution (e.g. Bernoulli)? If ys,r,n is an S × R × N tensor, how can it be added to bs,nus,n which, I assume is an S ×N matrix? What is the feature data which is used as input to the RF classifier, and what are the corresponding labels?

For each generated dataset, half of the subjects were set to diseased (1) and half were healthy (-1). Lower-case, non-bold symbols represent scalar variables throughout the whole paper, not matrices, and therefore they can be added. We realized that this paragraph could be confusing to the reader, and therefore we revised it in the new version of our paper.

Further comments on wording

Throughout the paper we softened the wording. However, to be clear, we continue to consider record-wise cross-validation when promising generalization to new subjects, not to be a subtle sub-optimality, but a major error that needs to preclude publication in journals. We would be very happy to have this statement be evaluated by professional statisticians (which we informally did).

References

Arlot, S., & Celisse, A. (2010). A survey of cross-validation procedures for model selection. Statistics surveys, 4, 40-79.

Hand, D. J. (2006). Classifier technology and the illusion of progress. Statistical science, 21(1), 1-14.

Rice, J. A., & Silverman, B. W. (1991). Estimating the mean and covariance structure nonparametrically when the data are curves. Journal of the Royal Statistical Society. Series B (Methodological), 233-243.

Xu, G., & Huang, J. Z. (2012). Asymptotic optimality and efficient computation of the leave-subject-out cross-validation. The Annals of Statistics, 40(6), 3003-3030.

Response to Reviewer 2

Comment: When first reading the title one could think that this is a junk paper. After reading the whole paper it turns out to be a serious and very well researched contribution. First hand it is highly recommendable to change the title as the paper does in no way deal with any Voodoo aspects and it is not explained what is meant with Voodoo, so either explain what is Voodoo machine learning or change the title.

We thank the reviewer for the helpful feedback and agree that the title was a bit misleading. Therefore, we changed it to “The need to approximate the use-case in clinical machine learning”. Although we want to add that this was a nod to a well-known preprint by Ed Vul who used the same voodoo verbiage. They also made him take it out as part of the review process.

Comment: The paper is readable and has a good structure; acronyms are also defined, here some suggestions on how to further improve the paper:
Check and revise formatting (whole paper)

We have formatted the paper to comply with the journal requirements. We will be happy to modify that based on specific suggestions of the editor and the reviewer.

Comment: Figures should be placed into the related paragraphs

We agree with the reviewer that it would be more appropriate to place figures into the related paragraphs. However, figures were placed at the end of the manuscript to comply with the journal formatting requirements during submission. We can include the figures in the text as well if the reviewer and the editor suggest. We agree that the world would be a better place if journals would switch to a figures-in-text format for submission.

Comment: Descriptions of the data sets have no references?

The reference to the publicly available human activity recognition dataset [19] has been updated with the correct URL. References to the code for generating the simulated dataset and the systematic review results are in the section “Availability of Supporting Data and Materials”, as required by the journal formatting guidelines.

Comment: Please mention the exact dataset(s) that were used in this research.

The database that we used was called “Smartphone-Based Recognition of Human Activities and Postural Transition Data Set”. We have now specified the exact name of this dataset in the section “Availability of Supporting data and materials”. In addition, we have provided the correct reference and link to the dataset. We also clarified that the generative model code can be downloaded by following the link mentioned in the same section.

Comment: on page 11, criteria 7) it says "Studies for which the CV type was "unknown"" - should this not read "not unknown"?

That is correct. We changed “unknown” to “not unknown”.

Comment: Figures 3 and 5 are blurry

We apologize for that. We believe this is a result of the PDF generation process following the upload of the manuscript, which is beyond our control. However, it is possible to download the original high-resolution figures by clicking on the link on the top of each page. We hope this helps addressing the issue.

Comment: Link to 'dataset' in reference 17 does not work (one "]" too much in URL)

The reference 19 (17 in previous submission) has been now updated with the correct URL.

Comment: Link in reference 17 only refers to the ML Repository of UCI, not a particular dataset

The link has been corrected, and now points to the specific dataset that we used.

Comment: General structure of the paper could be enhanced

We followed the specific format requirements of the journal. We will be happy to modify that based on specific suggestions of the editor and/or the reviewer.

Comment: Do not forget to revise the title (current title is misleading)
We did revise the title.

Comment: On page 3, line 59 you say: "In medicine, a great hope for machine learning is to automatically detect or predict disease ..."
Please mention here that in medicine, particularly in clinical medicine, automatic approaches greatly benefit from big data with many training sets. However, in the health domain, sometimes you are confronted with a small number of data sets, complex data, or rare events, where automatic approaches suffer of insufficient training samples. Here interactive Machine Learning may be of help, see:
Holzinger, A. 2016. Interactive Machine Learning for Health Informatics: When do we need the human-in-the-loop? Springer Brain Informatics, 3, (2), 119-131, doi:10.1007/s40708-016-0042-6, and an experimental example: Holzinger, A., Plass, M., Holzinger, K., Crisan, G., Pintea, C. & Palade, V. 2016. Towards interactive Machine Learning (iML): Applying Ant Colony Algorithms to solve the Traveling Salesman Problem with the Human-in-the-Loop approach. Springer Lecture Notes in Computer Science LNCS 9817. Heidelberg, Berlin, New York: Springer, doi:10.1007/978-3-319-45507-56.
On page 3, line 64-66, here is another recent clinical example of related work:

Donsa, K., Spat, S., Beck, P., Pieber, T. R. & Holzinger, A. 2015. Towards personalization of diabetes therapy using computerized decision support and machine learning: some open problems and challenges. In: Holzinger, A., Roecker, C. & Ziefle, M. (eds.) Smart Health, Lecture Notes in Computer Science LNCS 8700. Heidelberg, Berlin: Springer, pp. 235-260, doi:10.1007/978-3-319-16226-3_10.

We thank the reviewer for the suggested citations. We have included this aspect in the Discussion along with the two suggested citations (second to the last paragraph). We also added (Donsa et al, 2015) as another application to the Introduction (first paragraph).

Figure 1

Click here to download Figure Fig1.tif

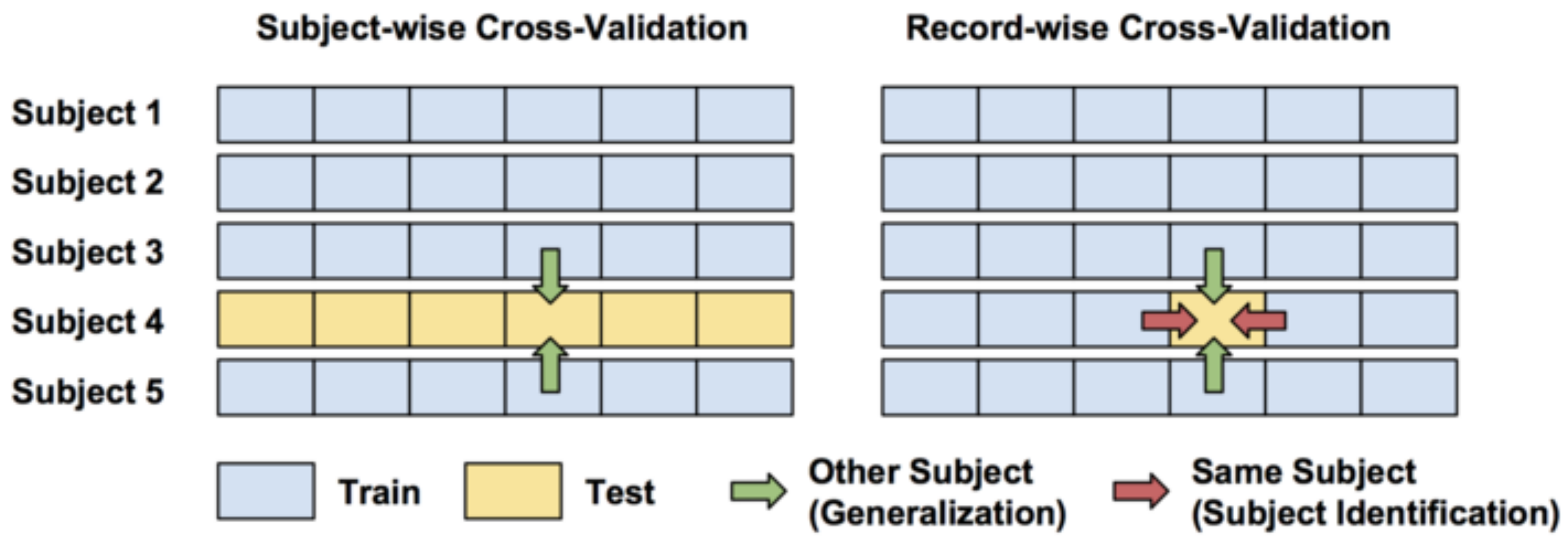

Figure 2

Click here to download Figure Fig2.tif

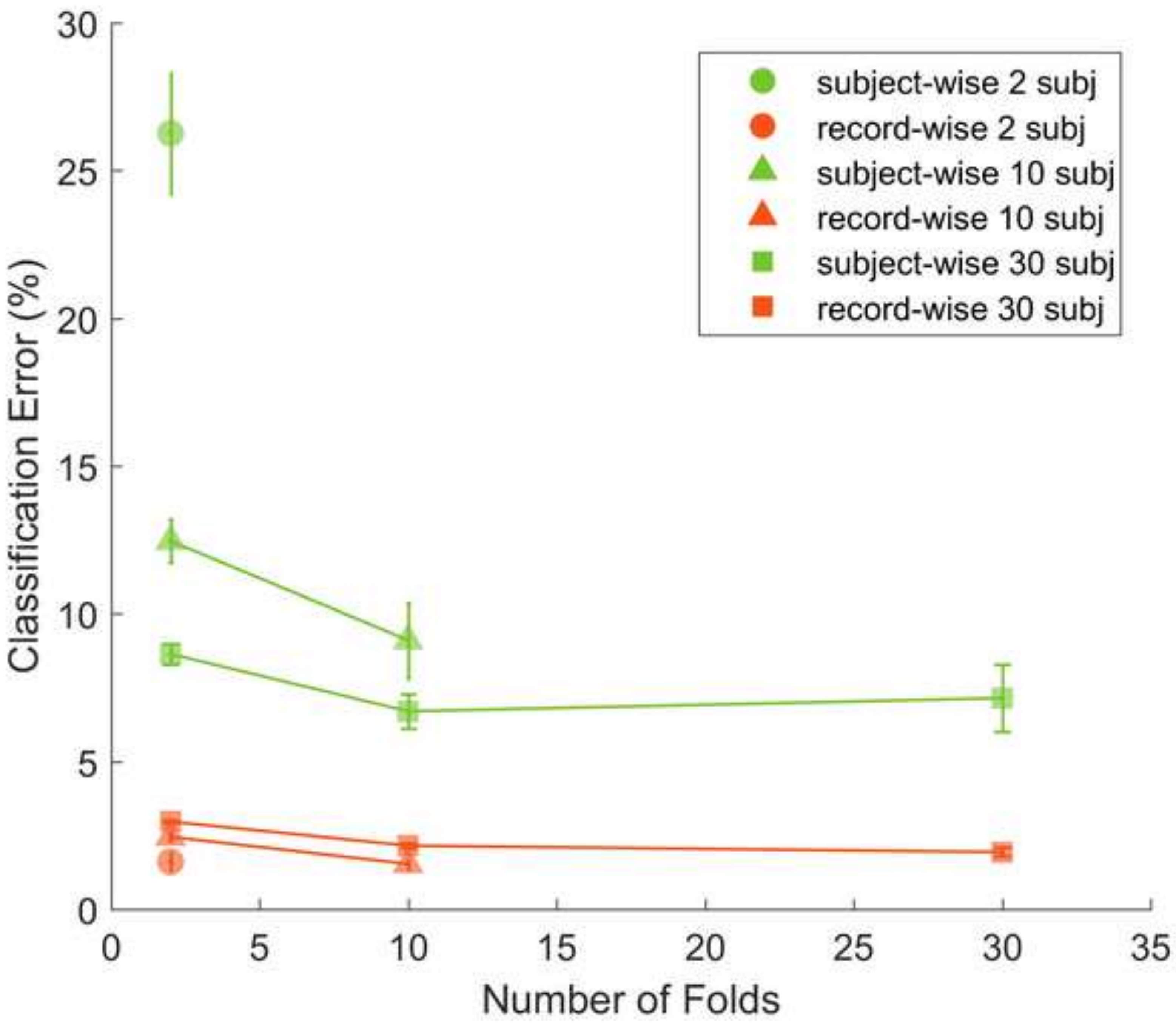

Figure 3 Click here to download Figure Fig3.tif

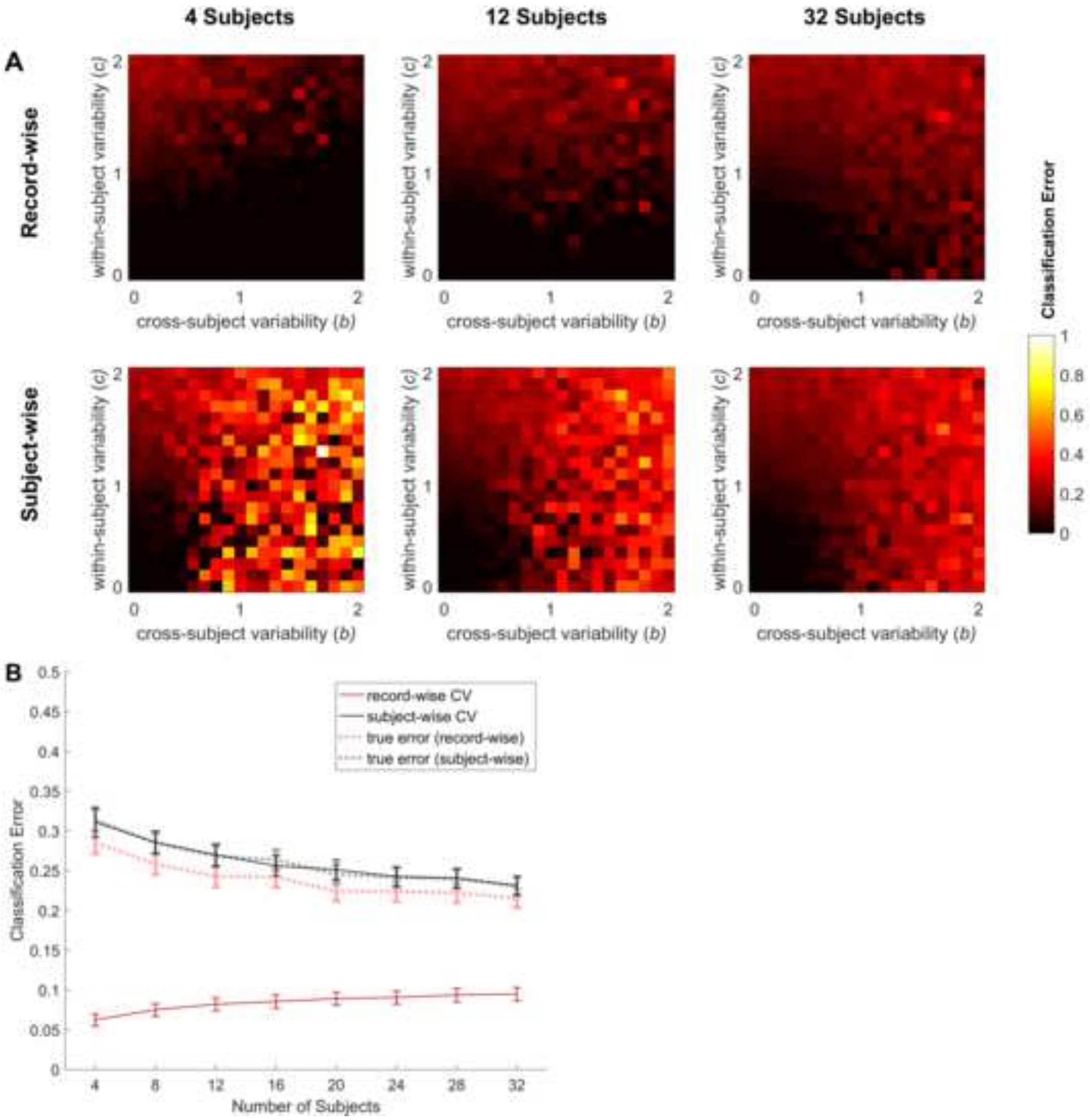

Figure 4

Click here to download Figure Fig4.docx

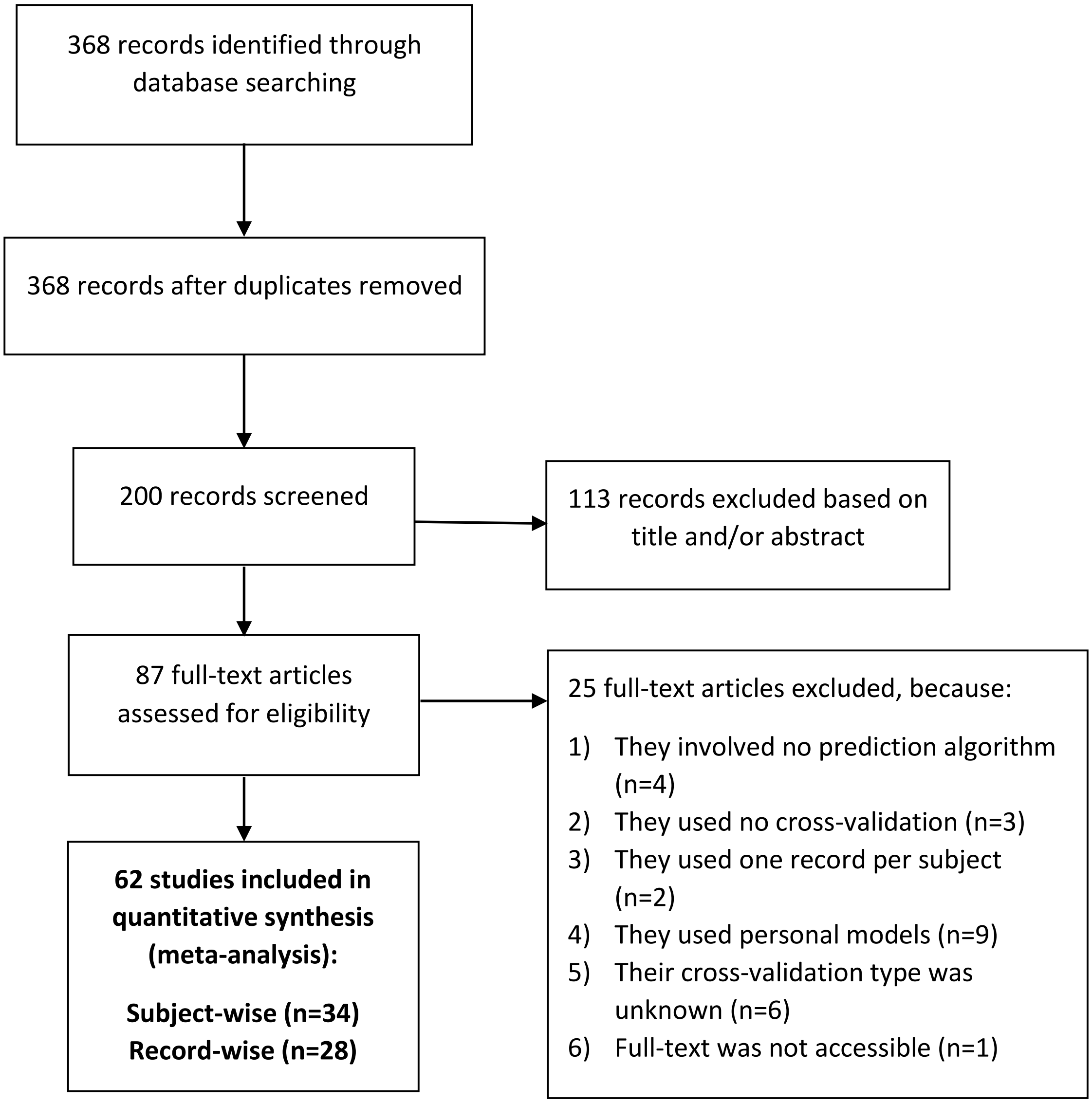

Figure 5

Click here to download Figure Fig5.tif

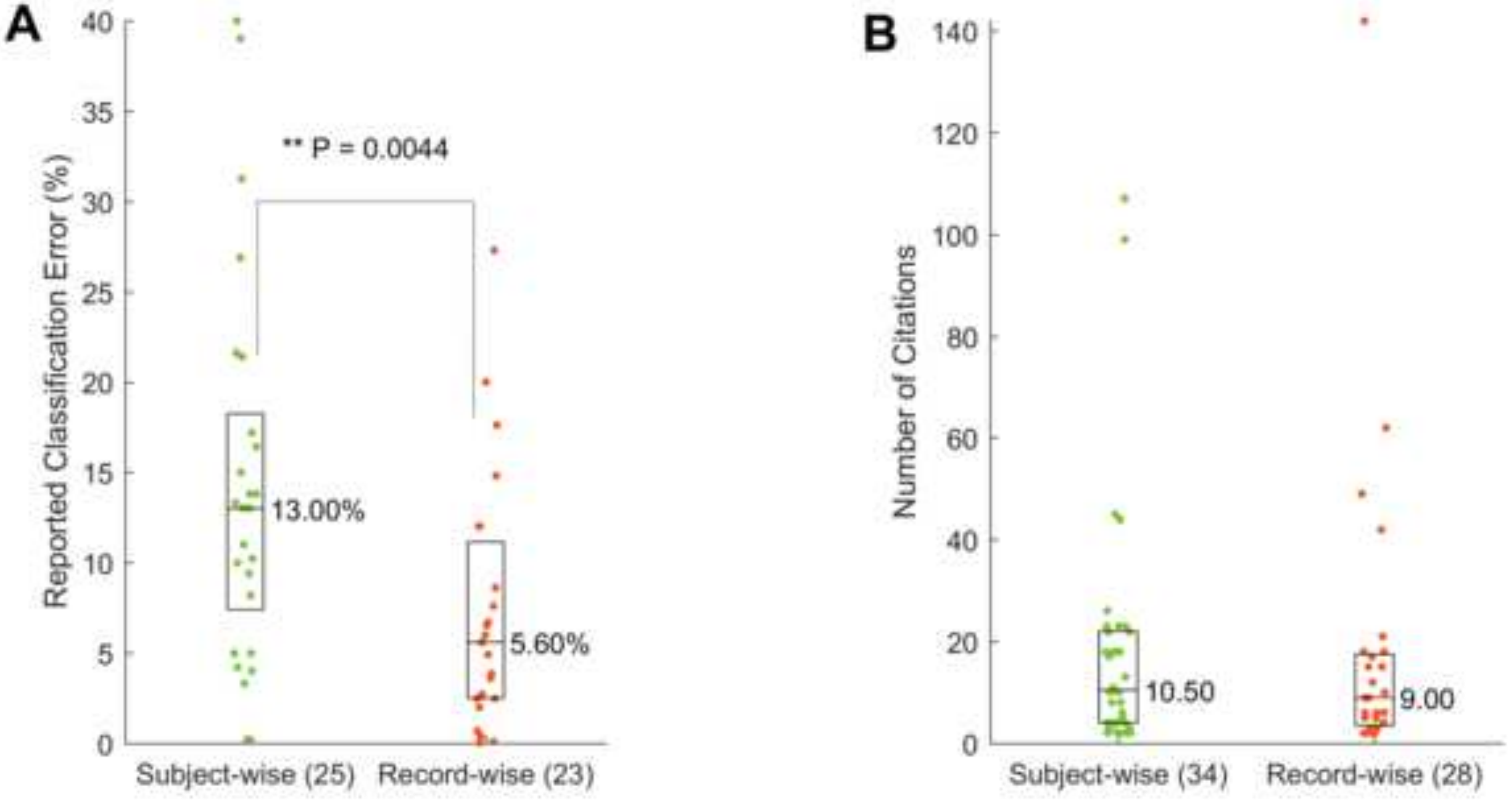

Supplementary Material - literature review results

Click here to access/download
Supplementary Material
review_results.xlsx

Click here to access/download
Supplementary Material
Manuscript_trackedchanges.docx
