# Supplementary material for: The need to approximate the use-case in clinical machine learning
Source: Gigascience. 2017 Mar 15;6(5):1–9. doi: 10.1093/gigascience/gix019 (PMC5441397; doi:10.1093/gigascience/gix019)
Supplement: Additional files: — Lonini_et_alPrepubHistory.pdf [file gix019_Lonini_et_alPrepubHistory.pdf]

Review of: "Voodoo Machine Learning for Clinical Predictions"

Max A. Little

August 3, 2016

1 Summary

In this interesting paper, Saeb et al. discuss the topic of cross-validation (CV) in studies that use smartphone or other wearable sensors to make clinical predictions using supervised machine learning classification methods. Based on evidence from analysis of publicly-available human activity recognition dataset, a systematic literature survey and a simulated mixed-effects model, they contend that the use of so-called "recording wise" CV systematically underestimates the true prediction error that would be expected if the prediction were applied to the population data, and that an alternative "subject-wise" CV method does not suffer from such systematic underestimation. They conclude that recording-wise CV should not be used in this context.

2 Overall review comments

Overall I find this paper very interesting. The investigation of the topic is worthwhile as CV is a key method for assessing generalization performance in the use of machine learning algorithms in general. We would ideally like to know that the method has been used such that the results can reliably lead to valid conclusions. Studies such as this, which seek to critically examine the use of such techniques are therefore valuable because they can help to improve on the quality of the science. Indeed, the idea that subject-wise CV should always be used in this context is commonly encountered "wisdom", but we need science not folklore so a systematic investigation of this point is necessary.

However, this paper has a fundamental flaw which can basically be summed up by saying that what the authors have uncovered is good evidence that the use of subject-wise CV *overestimates* the prediction error, rather than evidence that recording-wise CV underestimates error, as they contend. This completely turns the entire premise, and conclusions, of the paper on its head. The origin of this fundamental flaw is that the authors are not explicit (and possibly unaware of) the mathematical statistics which determine the conditions under which CV is a statistically valid procedure at all, and whether these conditions apply to the use of the method in each particular circumstance. (N.B. there are non-standard CV methods which rely upon different conditions but the authors are only addressing standard CV here).

To be more specific, the primary mathematical assumption of standard CV is that the data points, that is, samples from all the joint variables in the problem (e.g. features and outcome variables in this context) are drawn identically and independently (i.i.d.), from the joint population distribution over these variables [Arlot and Celisse, 2010]. When this is not true, CV is not a statistically valid procedure. More precisely, CV attempts to estimate the expected prediction error for the population distribution from a sample by splitting the sample into training and testing subsets, fitting the model to the training data, and estimating the prediction error on the testing subset. Now, any way of splitting an i.i.d. sample into subsets guarantees that these subsets themselves consist of i.i.d. realizations from the joint distribution over all the variables. Thus, the distribution of realizations in the training and

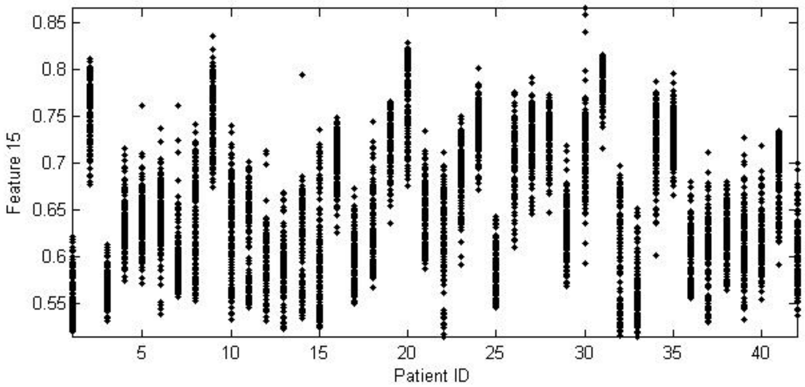

Figure 2.1: *Features from clinical datasets are often highly heterogenous, and do not share the same distribution across individuals. The problem with using subject-wise CV with this kind of data is that the assumption of standard CV, that the training and test sets come from the same distribution, is violated. This means that the results are statistically invalid, however, recording-wise CV does not suffer from this problem. Data from Tsanas et al. [2010]*

testing subsets are identical. When this is the case, it is valid to use the training subset to estimate model parameters and the test subset to estimate prediction errors, since the prediction error is being estimated on a different realization of these same random variables.

Indeed, the premise of hold-out testing, of which CV is an example, is that a model will tend to overfit if we use just one realization of a set of random variables to estimate the prediction error because, in this context, many machine learning models can be made as complex as desired and hence confound sampling variability for generalizable structure. So we must use a different realization to estimate errors. However, this procedure only works if we are testing and training on the same distribution, otherwise the model cannot be expected to make meaningful predictions on the testing data.

Now, returning to the context that the authors investigate in this manuscript where there are small numbers of participants and the studies are of behavioural and/or biological phenomena, the chance that the data is drawn i.i.d. from the population distribution, and hence statistically homogenous, is small. More realistically, and from my own experience, the data is highly heterogenous. For example, we often find examples of individuals whose within-subject variability is the same as the between-subject variability across the entire group. Furthermore, the data is often *clustered* by each individual, that is, we will find that variables tend to group in value within an individual (for a real-world example of this, see Figure 2.1). With such clustering, it is obvious that we cannot split the data by individual and expect that the variables in the training and testing subsets have the same distribution (see Figure 2.2 for an illustration on synthetic data). In this situation, subject-wise CV is not statistically valid because the primary i.i.d. assumption is violated.

What is the practical effect of trying to estimate prediction errors using subject-wise CV in this subject-wise clustered-variable situation? That of course depends upon many factors, including the specifics of the model and the data. But for illustration, with a simple polynomial regression model applied to clustered Gaussian mixtures, the effect of this clustering causes subject-wise CV to grossly overestimate the prediction error and thus select a model with underfitted complexity, because of the distributional mismatch (see Figure 2.3 and the Appendix for details of the synthetic model). In this situation, and as anticipated, recording-wise CV accurately estimates the population prediction error and the correct model complexity is selected. Recording-wise CV works here precisely for the reasons that the theory suggest [Arlot and Celisse, 2010]. By contrast, splitting the data by subject *guarantees* distributional mismatch, the model is only exposed to a small part (an essentially constant part) of the overall structure in the data (which is a quadratic curve), and erroneously underfits to that small part. The model cannot be expected to do anything other than underfit since it is not exposed to

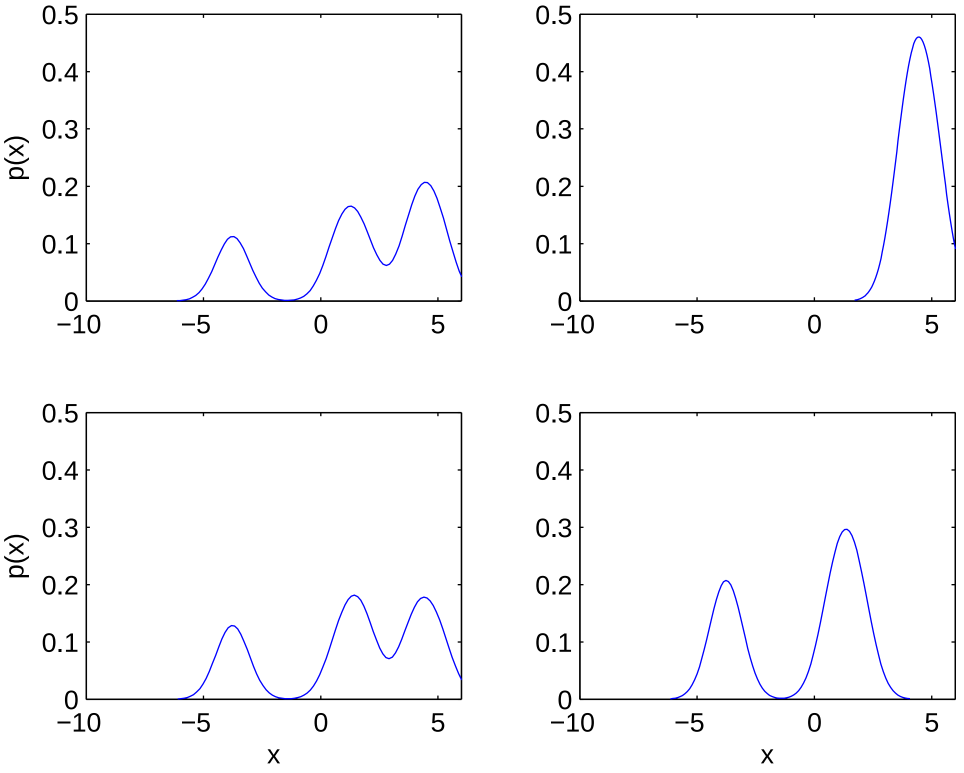

Figure 2.2: *The distribution of the covariate X for the example train (top row) and test sets (bottom row) for the basic non-stratified (e.g. recording-wise, left column) and hidden (mixture component indicator) covariate-stratified (e.g. subject-wise right column) cross-validation schemes. As can be seen, while the data is drawn i.i.d. from a mixture distribution, the non-stratified (reording-wise) scheme leads to indistinguishable distributions for training and test sets, whereas, in the stratified (subject-wise) case, the train/test distributions are radically different. Therefore, covariate stratification (i.e. splitting by subject ID) violates an essential assumption of cross-validation. See Appendix for further details.*

the whole structure in the data which is only visible if a subset of data across all subjects is used in training (see Figure 2.3). In my experience the effect of covariate clustering such as this is to cause systematic underfitting; indeed concurring with the findings of the systematic literature review in this manuscript.

In the main, I do agree with the authors' stated aim of improving the quality of the science in the discipline. I also agree that addressing the problems of overfitting are clearly important in this discipline, and that there are some rather easy of ways of making confounded "predictions" using overly complex machine learning methods and small datasets (which the paper discusses, but see specific comments below), and also given how easy it is to fool oneself with pernicious problems such as data leakage [Kaufman et al., 2011]. But a huge amount is already known and written about this (e.g. read Hand, 2006) and this paper does not get us further towards this goal because of the fundamental misapprehension about the mathematical foundations of CV. Nor does the excessive hyperbole (see specific comments below) and the overblown polemical rant (that belongs on the editorial opinion pages, or Twitter, and not in a scientific article). The end result of the paper as written will just be to further confuse what is an already complex and subtle topic.

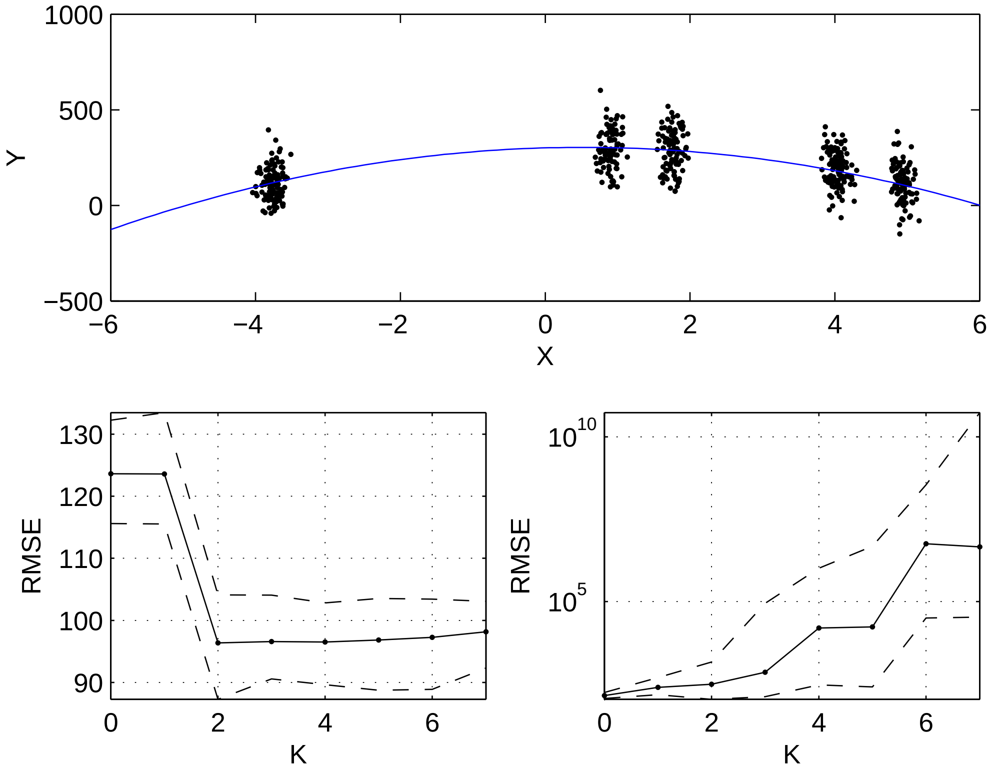

Figure 2.3: *A simple quadratic model ($Y = 300.7 + 10.6X - 10.1X^2$) for Y given X (top panel, blue curve), where the X data ($N = 500$ samples) is drawn from a Gaussian mixture model ($M = 5$ components), with mixture component indicators Z. The mixture components have standard deviation $\sigma_X = 0.1$. The Y data (top panel, black dots) has additive zero-mean Gaussian with standard deviation $\sigma = 100$; thus the population prediction error is 100. Non-stratified (e.g. recording-wise) cross-validation (uniformly random, independent partitioning, 50% train/50% test, 100 repetitions) leads to good cross-validated prediction error estimates (fitted by least-squares) and selects the correct, quadratic model $K = 2$ (bottom left panel, dotted solid line is the mean prediction error, dashed lines are the minium and maximum prediction error over all repetitions), whereas, resampling by stratifying on the indicator Z (e.g. patient ID) leads to very substantially exaggerated prediction error and selection of the underfitted model $K = 0$ (bottom right panel). The cause of the problem is that stratification causes the violation of the essential assumption of cross-validation that the training and test sets are matched in distribution. See Appendix for further details.*

While empirical investigations are undoubtedly interesting and valuable in their own right, what is needed, rather than attempting to draw conclusions from inevitably limited empirical evidence alone, is to develop tools for, and simultaneously investigate, testing the applicability of the fundamental mathematical statistics of techniques such as CV. For example, how would we test that training and test sets agree in distribution? How would we test that the data is really i.i.d.? Are the training and test sets truly independent? What are the practical limitations of CV, and what are the alternatives? The issues can be subtle and quite complex [Kubota et al., 2016]. This is similar to the program that classical statistics pursues of e.g. testing for normality before using the t-test, for example. In my opinion, another, similarly valuable direction, is to raise awareness of more appropriate techniques which can be used in the situation where distributional mismatch occurs, e.g. covariate shift [Sugiyama et al., 2007, Shimodaira, 2000], domain adaptation [Blitzer et al., 2008], methods for class imbalance, sample selection bias and dealing with data drift [Hand, 2006].

3 Specific comments

1. Title. This doesn't make any sense. What do the authors mean exactly by "voodoo machine learning" and "clinical predictions"? The title needs to be changed to actually describe the topic of the paper.

2. P2, L36: "however, several cross-validation methods exist and only some of them are statistically meaningful." What exactly is meant by this? Yes, there are many CV methods [Arlot and Celisse, 2010, Shimodaira, 2000, Sugiyama et al., 2007], but they are all, by definition "statistically meaningful". So, this statement needs to be corrected.

3. P2, L41: "... massively overestimates ...". This is just hyperbole, and (see above), this is not a correct statement either. What has been observed is that there is a systematic discrepancy between recording-wise and subject-wise CV, and this is all that can be claimed on the basis of the evidence. This statement needs correction.

4. P2, L42: ".. this erroneous method ...". In theory there should be no difference between recording-wise and subject-wise CV if the data is truly i.i.d. so recording-wise is not "erroneous" because it is just plain CV. In fact, subject-wise can be erroneous if the data is subject-wise clustered, as is often the case. This statement therefore needs correction.

5. P2, L46: "... proper methods ..." What exactly is meant by "proper" here? This statement needs clarification.

6. P4, L80: " ... the proper form of CV is subject-wise: here the training and test sets contain records from different subjects; therefore, the performance 82 of the classifier is measured on a new subject whose data has not been used for training (Figure 1A)." This is the often repeated folklore about subject-wise CV used in this context. From the discussion above, it should be clear why there *cannot be any difference* between subject-wise and recording-wise CV, *if the data are truly i.i.d. as required by the assumptions of the standard CV algorithm [Arlot and Celisse, 2010].* Therefore CV, if applied correctly, cannot be used to draw any special, application-specific conclusions about how the machine learning algorithm might perform when deployed in a particular practical configuration. If, on the other hand, as I suspect, what the authors are claiming is that they expect the training, test and deployment data to differ in distribution, then standard CV cannot be used to assess the expected prediction error for the population. Instead, one should turn to the appropriate methods such as covariate shift [Shimodaira, 2000] or domain adaptation [Blitzer et al., 2008]. This whole sentence needs to be revised in order to accurately reflect these facts about CV.

7. P4, L85: "In this way, the machine learning algorithm can find an association between unique features of a subject (e.g., walking speed) and their clinical state, which automatically improves its prediction accuracy on their test data. As a consequence, the record-wise CV method can significantly overestimate the predicted accuracy of the algorithm." This is a vague statement. What the authors are referring to, I suspect, are examples of confounds which arise due to the interaction between certain machine learning methods and particular data sets. For example, where (a) an overly complex machine learning method is used, (b) the data has only a small number of heterogenous subjects, and (c) the features for each subject have sufficiently small variability and are all clustered within a region of the feature space which is unique to each individual. Under these special circumstances, it is possible for the identity of each individual to be mapped onto the clinical outcome in a way which confounds the prediction of health status from the data. But, there could be all sorts of confounds like this, for example, it is possible for age to be inadvertently mapped onto the clinical outcome, because age is the primary variable for predicting pretty much *any* adverse health outcome (just ask any health insurer!) It might also be possible to infer this indirectly, for instance, since some feature might be highly dependent

upon age and so become a ‘proxy variable’ for age. The authors want to turn this statement about confounds into a statement about how recording-wise CV is the problem and if only we used subject-wise CV the problem would go away. But actually the problem is the confound and the fact that without understanding the machine learning method we are using and the nature of the data to which it is being applied, one cannot use CV to fix the confound, because in these circumstances the model is useless anyway. The fact that subject-wise CV seems empirically to produce lower prediction error estimates in some cases does not mean that the confound has been fixed and by using it we now have an accurate estimate of the population prediction error. If instead, what is being argued is that you can use different kinds of splitting of the data to try to diagnose various confounds rather than actually estimate population prediction errors, then the authors should state that in the paper. But they should not make a claim that this means they are applying CV as theoretically intended. So, this paragraph is misleading and needs to be rewritten.

8. P4, L93: “The problem is, that if the algorithm can (implicitly) detect the identity of the person based on the features, it can automatically also “diagnose” the disease.” As in the point above, this is a misleading sentence, which implies that recording-wise CV is the problem where in fact the problem is due to a hopelessly confounded machine learning algorithm applied to data which supports that inadvertently confounded prediction. The sentence is misleading and needs to be revised or removed.

9. P4, L97: Paragraph beginning “As this statistical problem ...” What do the authors actually mean here by saying the problem is “statistical”? There is no mention of random variables for a start, and all the ensuing discussion is essentially about a purely deterministic scenario. Also, see the discussion in point 7: the simplified situation envisaged here is just another example of the confounding discussed there. So, as with the above, this paragraph is misleading and needs to be rewritten.

10. P5, L110: “... demonstrate the potential threat to the clinical prediction literature when the wrong CV methods are used,” This is hyperbole: what is meant by a “potential threat” and what is meant by the “wrong CV methods”? See the points above. This sentence needs to be rewritten.

11. P5, L112: “... CV yield dramatically different results, with record-wise CV massively overestimating the accuracy.” This is just yet more hyperbole. What does “dramatically different” or “massively overestimating” mean, precisely? See also points above about the theoretical basis of CV. Needs rewriting.

12. P5, L116: “... quantify the prevalence of this problem ...” What problem exactly? See points above about the theoretical basis of CV.

13. P5, L118: “... along with their classification accuracies and number of citations.” I do not understand why the number of citations is of any relevance here. What exactly are the authors trying to claim, that articles which use recording-wise CV should not be read and cited? Presumably articles can be cited for many other reasons than just the use or not of one specific technique. This needs further clarification.

14. P7, L157: “... record-wise CV significantly overestimated” More hyperbole. See points above. This needs to be rewritten.

15. P7, L163: “... record-wise CV significantly inflates” Further hyperbole. See points above. Needs rewriting.

16. Simulated Clinical Prediction. This is an interesting experiment but it is not sufficiently well described to allow replication. Are the +1/-1 labels which indicate healthy/disease status generated at random? If so, what is their distribution (e.g. Bernoulli)? If $y_{s,r,n}$ is an $S \times R \times N$

tensor, how can it be added to $b_{s,n}u_{s,n}$ which, I assume is an $S \times N$ matrix? What is the feature data which is used as input to the RF classifier, and what are the corresponding labels?

17. P10, L223: "This is because as we increase the number of subjects, it becomes harder for the record-wise classifier to identify subjects based on their data, thereby losing its advantage over the subject-wise method." There needs to be a much more detailed and systematic investigation and explanation for how it is that the interaction with the RF classifier and the data produced here leads to confounded predictions based upon subject identity alone. Otherwise, this statment is just supposition. For example, one might start by investigating the diversity of subjects at each RF node.

18. P14, L310: "... exhibiting an inflated performance yielded by record-wise CV." More likely, this is underestimated accuracy by subject-wise CV, or at least, we cannot rule out that entirely contrary explanation. See points above. This sentence will obviously need revision.

19. P14, L311: "Therefore, improper cross-validation has led to dramatically "better" results." Further hyperbole. What exactly is meant by "improper" here? This sentence needs revision.

20. P14, L313: "Therefore, whether a paper used the correct or wrong CV method did not affect the perceived impact of that paper in the field." I hope that the authors are not drawing the overblown implication that 50% of the literature in this discipline is so wholly erroneous that it should not be read or cited ... I think the authors would need extraordinary evidence for that extraordinary conclusion. Again: what is the "correct" or "wrong" CV method? Furthermore, why exactly would the choice of CV method "affect the perceived impact" of the paper? Additionally, I do not see why citations alone are a reflection of "perceived impact" of a paper, and in fact I do not really know what is meant precisely by "perceived impact" either! All of these points need referencing and further explanation, although I do not know what would be the relevant discipline here (bibliometrics, perhaps?)

21. Discussion: This section is full of opinion pieces, and ends with an overblown polemical rant about "(im)proper training' of researchers creating "fallacious findings" and generating "erroneously positive results" which "threaten progress". The authors seem to have gone completely off the rails with statements which contribute nothing to the interpretation of the evidence. These need to be removed. The paragraph on hyperparameter tuning is vague, and has little to do with the topic and nothing to do with the evidence presented in the paper, and so should be removed. Similar comments apply to the section on reproducibility which again is not directly related to the point of the paper. Regarding the opinion pieces, this entire section needs to be rewritten to make statements which can be justified solely on the basis of the evidence presented and the theoretical foundations of CV. I will not list every example, but e.g. use of adjectives such as "wrong" or "correct" and especially "meaningful/meaningless" are red flags in this regard.

4 Appendix: simulating subject-wise CV: clustered hidden covariates in regression

We will consider the simple situation where the data $\mathcal{D} = (x_n, y_n)_{n=1}^{N}$ is generated by a univariate polynomial regression model $Y = \sum_{k=0}^{K} a_k X^k + \epsilon$ with parameter vector a. The noise term ϵ will be i.i.d. $\mathcal{N}(0, \sigma^2)$. The application is twofold: we wish to estimate the out-of-sample prediction error, and use model selection to choose the optimal order of the model, the degree of the polynomial, K. We will assume that the actual data is generated by $K = 2$ (the quadratic model). Let us also assume that the data X is sampled i.i.d. from a Gaussian mixture distribution with M components, $f(x) = \frac{1}{M} \sum_{m=1}^{M} \mathcal{N}(\mu_m, \sigma_X^2)$.

Now, it is easy to see that the joint density X, Y may have several distinct modes, but note, the data $\mathcal{D}$ is still i.i.d. Thus, any uniformly and independently random partition (that is, independent of any other variables) of the data for CV purposes will ensure that the training and test sets are still drawn from the joint distribution over X, Y: this is the crucial "identically distributed" part of i.i.d (Figure 2.2, left panels). We can see that the normal cross-validated model selection works exactly as expected, picking out the $K = 2$ model (Figure 2.3, bottom left panel).

Next, we will rewrite the mixture model above such that we can partition the data pairs (x_n, y_n) into those which belong to the same cluster. We can do this by introducing the *latent* or *"hidden" variable* Z which is discrete taking on the values $\{1, \ldots, M\}$ with categorical distribution and uniform parameters. We can now redefine the Gaussian mixture for X in terms of this latent variable, i.e. $f(x|z) = \mathcal{N}(\mu_z, \sigma_X^2)$. This means that the joint distribution $f(x, z) = \frac{1}{M}\mathcal{N}(\mu_z, \sigma_X^2)$ and so by summing over $z = 1, \ldots, M$ we arrive at our original mixture distribution $f(x)$.

The next step is to assume that the CV method has access to the data for Z, e.g. we have $(z_n)_{n=1}^{N}$ available. Now, instead of partitioning the data independently at random to form the train/test sets, we will ensure that any data (x_n, y_n) where $z_n \in T$ goes into the training set. Here T is just a set containing some values from $1, \ldots, M$. The rest of the data where $z_n \notin T$, goes into the test set. Thus, the partioning into train/test subsets now depends upon the hidden variable Z.

We can see that in implementing this new method, the distribution over train and test sets differ substantially (Figure 2.2, right hand panels). Now, the prediction error is significantly overestimated for the polynomial model which actually generated the data (Figure 2.3, bottom right panel). Partitioning this way also selects the wrong model, $K = 0$, which is underfitted. In fact the $K = 0$ model is just making predictions using the *mean* of the training data for Y alone, which entirely ignores the covariates. This is the most extreme underfitting possible in this model.

What has gone wrong? Since we have allowed the CV partitioning to be dependent upon the data Z rather than to be entirely independent, the X values in the training set are each drawn from one of the mixture components in the set T, $\mathcal{N}(\mu_{z\in T}, \sigma_X^2)$, and the samples in the test set are drawn from the remaining mixture components, $\mathcal{N}(\mu_{z\notin T}, \sigma_X^2)$, instead. So, the distributions of the training and test sets are quite different, and the assumption of CV that the training and test sets are matched, which flows from the i.i.d. assumption, is violated. Note that the *original* data are still i.i.d. from the population distribution – but the training and test sets have become *stratified* which causes significant mismatch between their distributions. Clearly, stratified CV is meaningless in this situation because the crucial underlying assumption that the test and training data are from the same underlying distribution, no longer holds. The effect of this mismatch interacts with the asymptotic extremes of the polynomial giving predictions which are substantially wrong.

References

Sylvain Arlot and Alain Celisse. A survey of cross-validation procedures for model selecion. *Statistics Surveys*, 4:40–79, 2010.

John Blitzer, Koby Crammer, Alex Kulesza, Fernando Pereira, and Jennifer Wortman. Learning bounds for domain adaptation. *Advances in Neural Information Processing (NIPS) 2008*, 2008.

David J. Hand. Classifier technology and the illusion of progress. *Statistical Science*, 21(1):1–14, 2006.

Shachar Kaufman, Saharon Rosset, and Claudia Perlich. Leakage in data mining: formulation, detection and avoidance. *KDD'11*, 2011.

K.J. Kubota, J.A. Chen, and M.A. Little. Machine learning for large-scale wearable sensor data in parkinson disease: concepts, promises, pitfalls and futures. *Movement Disorders*, 2016. (in press).

Hidetoshi Shimodaira. Improving predictive inference under covariate shift by weighting the log-likelihood function. *Journal of Statistical Planning and Inference*, 90:227–244, 2000.

Masashi Sugiyama, Matthias Krauledat, and Klaus-Robert Müller. Covariate shift adaptation by importance weighted cross validation. *Journal of Machine Learning Research*, pages 985–1005, 2007.

A. Tsanas, M.A. Little, P.E. McSharry, and L.O. Ramig. Accurate telemonitoring of parkinsons disease progression by non-invasive speech tests. *ieee Transactions on Biomedical Engineering*, 57 (4):884–893, 2010.

Review Andreas Holzinger (previous version of the manuscript)

When first reading the title one could think that this is a junk paper. After reading the whole paper it turns out to be a serious and very well researched contribution. First hand it is highly recommendable to change the title as the paper does in no way deal with any Voodoo aspects and it is not explained what is meant with Voodoo, so either explain what is Voodoo machine learning or change the title.

The paper reports on two different types of cross-validation, namely record-wise and subject-wise cross-validation. The authors found out that the prediction precision is mostly overestimated in the "Human Activity Recognition" data set and is underestimated in the "Simulated Clinical Prediction" data set. The "Human Activity Recognition" data set contains 6 activities (walking, sitting, standing, laying down, stair climbing up and down) and the data is generated from the sensors of a smartphone. The "Simulated Clinical Prediction" data set is generated with a generative model which includes cross-subject and within-subject variabilities. Furthermore, the authors carried out a literature review with Google Scholar to show how often papers are published in this domain. Papers are compared among the number of citations, classification accuracies and CV methods. CV's outcome could be incorrect if the hyper-parameters are not well adjusted. If the data set is split into a training and a validation set, you must be careful with the subjects by using record-wise and subject-wise cross-validation.

Concerning the originality of this paper: This paper uses data sets, studies and algorithms which already exist. The novel aspects include the comparison of various studies and the prediction precision with record-wise CV is mostly overestimated. Note that the paper has already been uploaded at http://biorxiv.org/content/early/2016/06/19/059774.

The paper is readable and has a good structure; acronyms are also defined, here some suggestions on how to further improve the paper:

* Check and revise formatting (whole paper)
* Figures should be placed into the related paragraphs
* Descriptions of the data sets have no references?
* Please mention the exact dataset(s) that were used in this research.
* on page 11, criteria 7) it says "Studies for which the CV type was "unknown"" - should this not read "not unknown"?
* Figures 3 and 5 are blurry
* Link to 'dataset' in reference 17 does not work (one "]" too much in URL)
* Link in reference 17 only refers to the ML Repository of UCI, not a particular dataset
* General structure of the paper could be enhanced
* Do not forget to revise the title (current title is misleading)
* On page 3, line 59 you say: "In medicine, a great hope for machine learning is to automatically detect or predict disease ..."
Please mention here that in medicine, particularly in clincial medicine, automatic approaches greatly benefit from big data with many training sets. However, in the health domain, sometimes you are confronted with a small number of data sets, complex data, or rare events, where automatic approaches suffer of insufficient training samples. Here interactive Machine Learning may be of help, see: Holzinger, A. 2016. Interactive Machine Learning for Health Informatics: When do we need the human-in-the-loop? Springer Brain Informatics, 3, (2), 119-131, doi:10.1007/s40708-016-0042-6, and an experimental example: Holzinger, A., Plass, M., Holzinger, K., Crisan, G., Pintea, C. & Palade, V. 2016. Towards interactive Machine Learning (iML): Applying Ant Colony Algorithms to solve the Traveling Salesman Problem with the Human-in-the-Loop approach. Springer Lecture Notes in Computer Science LNCS 9817. Heidelberg, Berlin, New York: Springer, doi:10.1007/978-3-319-45507-56.
On page 3, line 64-66, here is another recent clinical example of related work:
Donsa, K., Spat, S., Beck, P., Pieber, T. R. & Holzinger, A. 2015. Towards personalization of diabetes therapy using computerized decision support and machine learning: some open problems and challenges. In: Holzinger, A., Roecker, C. & Ziefle, M. (eds.) Smart Health, Lecture Notes in Computer Science LNCS 8700. Heidelberg, Berlin: Springer, pp. 235-260, doi:10.1007/978-3-319-16226-3_10.

More deeper review, concerning the experiments:

1. A Simulation on a Human Activity Recognition dataset was conducted to show differences in subject-wise vs. record-wise prediction accuracy: 30 subjects performing 6 activities; random forests for classification - the ensemble decided by averaging over the decision tree predictions. The thought behind this strategy was that a random forest has fewer parameters to tune because each tree only sees a subset of the data and is thus less prone to overfitting.
1. First the authors were interested in the classifier accuracies when using subjectwise vs. record-wise CV. They used different numbers of subjects as well as folds and
showed that record-wise CV consistently overestimates accuracy.
2. In a second step a generative model was used to create a model combining crosssubject and within-subject variabilities to generate an observed record and concluded that
cross-subject variability had a much greater effect on subject-wise CV than record-wise CV (as the latter already implies knowledge about cross-subject variability)
2. In the second part of the paper, a literature study was undertaken to estimate what percentage of studies actually use record-wise CV; papers were searched for by keywords (including 'cross validation'). The selection process yielded 62 papers from an input set of 369 papers found by google scholar; out of those, 34 used subject-wise CV and 28 used record-wise CV - about 45%. The subject-wise studies showed a classification error of more than twice the error produced by record-wise studies, which supports the main statement of the paper.
The authors further explain that arbitrarily adjusted hyper-parameters are an additional source of prediction accuracy overestimation. Although only one dataset was used in the practical simulation, they argue that the theoretical underpinnings for their suggestion

should generate to any (clinical) dataset.

Overall, it is an interesting paper, relevant and nice to read, with some adaptations it could be very valuable for the readers of this journal and be of support for the international research community.

Response to Reviewer 1

First, we want to thank the reviewers for participating in this non-anonymous review process. Their responses have made our paper dramatically stronger. However, there are also a range of issues that we cannot agree with as we will detail below.

Records are not *i.i.d.*

The core of Dr. Little's argument is that we want our training and test subsets be identically-distributed in order to have maximally similar distributions, and since subjects have different distribution of covariates, we should sample from all subjects in training and test subsets to get similar distributions:

> data.
>
> Now, returning to the context that the authors investigate in this manuscript where there are small numbers of participants and the studies are of behavioural and/or biological phenomena, the chance that the data is drawn i.i.d. from the population distribution, and hence statistically homogenous, is small. More realistically, and from my own experience, the data is highly heterogenous. For example, we often find examples of individuals whose within-subject variability is the same as the between-subject variability across the entire group. Furthermore, the data is often *clustered* by each individual, that is, we will find that variables tend to group in value within an individual (for a real-world example of this, see Figure 2.1). With such clustering, it is obvious that we cannot split the data by individual and expect that the variables in the training and testing subsets have the same distribution (see Figure 2.2 for an illustration on synthetic data). In this situation, subject-wise CV is not statistically valid because the primary i.i.d. assumption is violated.
>
> [illegible]

There clearly is variability across subjects – that is the point of the entire paper. But we cannot at all agree with the statistical conclusions of Dr. Little. We see a great deal of statistical confusion in this argument and we want to step through the logic very clearly.

The definition of a good statistical approach is that it estimates the relevant variables. In the context of machine learning in medicine, by definition, the relevant variable, the variable that doctors want to know about, is if we would implement the ML approach and then recruit more patients, how well would it do on these patients. That is what the real world use and that is what investors, doctors and other scientists want to use when evaluating how well a system works. So, let us formulate that very clearly and then come back to the *i.i.d.* issue. We want to estimate how well our machine learning system would work on a new batch of patients. Good performance means having an estimator that is minimally biased and has low variance. In other words, if we read an ML paper that shows great performance, the reader wants to take away that it will work in the real life scenario, which means it will work in a situation with new patients.

Now, let us get back to Dr. Little's comments. There are two potential *i.i.d.*s involved here, and his argument is confusing the two of them. First, when proper recruitment procedures are used, then subjects are in good approximation drawn *i.i.d.* from the overall population of subjects. In human subject studies, this means that we need to have a large and representative sample of the population. However, in such a scenario, records are definitely non-*i.i.d.* In fact, as Dr. Little points out, records from the same subjects tend to be highly correlated to one another and are thus not independent. The point that we will predict worse by excluding records from the same subject is, in this context, a truism. But the fact that different subjects are distinct is the central problem. Also, the fact that records are not *i.i.d.*, even if subjects are, is an important fact of life. This is exactly the effect that makes record-wise cross-validation underestimate the errors as we will show clearer in the revised version of the paper.

The violation of *i.i.d.* across records is exactly what produces the erroneously low error rates of the record-wise strategy. Some of the variance, as Dr. Little rightfully points out, relates to the subject. The records from the same subject are not independent. They are correlated because they come from the same subject. And hence, whatever label or variable is estimated from the samples will effectively, improperly utilize subject-specific features to predict the disease. For a formal justification of the optimality of leave-subject-out cross-validation we want to refer the interested reader to Xu and Huang (2012). So to be clear, the leave-one-subject-out cross-validation has been proven to be correct. The record-wise cross-validation in this context violates the independence assumption, and without independence, it overestimates the prediction accuracy (and the new figure 3b very clearly shows this).

A detailed comparison with the ground truth

The goal of machine learning is to develop algorithms that not only work for the specific dataset they have been trained or cross-validated on, but to generalize to new data. But what does new data mean in clinical prediction problems? It depends on what the algorithm is developed for. If the algorithm is developed specifically for one subject, new data means new records from the same subject. This falls into the category of *personal* models which were excluded from our study. However, if the goal is diagnosis, meaning that we want to develop *global* algorithms that can predict a clinical outcome based on the records from a new subject, new data means new subjects. Therefore, the true prediction error can be defined as the prediction error of the algorithm on new subjects from the same population, which have not been used for cross-validation. The goal of cross-validation is to estimate this error.

In our simulation study in the paper (Simulated Clinical Prediction), we can estimate the true prediction error by generating an additional dataset, that is generated using the same generative model. The prediction error of the models that are trained and cross-validated on the main dataset is evaluated on this new dataset and will serve as the true prediction error. Then, we can compare the prediction errors estimated by cross-validation on the main dataset to the true error.

Here, we generated new data for 10 subjects and considered that as the new dataset. We evaluated each of the trained models in Figure 3B on this dataset. As the results show, the prediction error calculated using subject-wise cross-validation is a much better estimator of the true prediction error, and the record-wise method is massively underestimating it. Above all, besides obvious minor biases, we can see how subject-wise CV correctly tracks the variables of interest while record-wise CV does not.

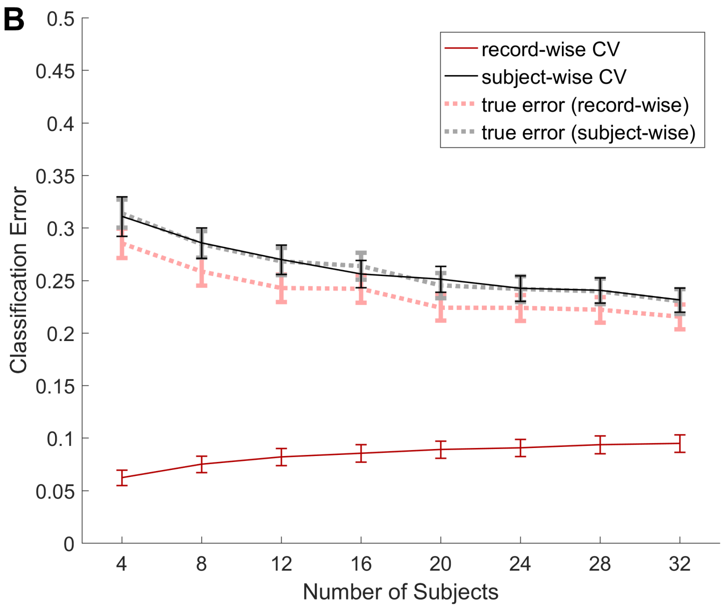

Figure 1. (Figure 3B in the new manuscript). Classification error on a simulated dataset for record-wise and subject-wise cross-validation (CV) methods, along with the true prediction errors computed on a new dataset, as a function of number of subjects. The number of features is set to 10. The mean and the 95% confidence interval of the classification error is shown.

The provided simulations (Figure 2.3) were grossly misleading

We reproduced the simulation that the reviewer has done in Figure 2.3 of the response letter. While we got the same results as the reviewer for record-wise RMSE results (Fig 2A), the results for subject-wise cross-validation depended on how many subjects were used for training and how many for test, since there were only 5 subjects in the dataset. We can only replicate the results in Figure 2.3 if we used 1 subject to train and the remaining 4 for test (Fig 2B). This, however, is an extremely unusual way to do subject-wise cross-validation. In fact, it makes no sense whatsoever, as generalizing from a single subject to a population is generally impossible. When we have such a small dataset, the common way to do subject-wise cross-validation is leave-one-out, also called jackknife. And in fact, if we cross-validate using the leave-one-subject-out method, the resulting RMSE looks like Fig 2C, which is very different from Fig 2B and what the reviewer has shown in his response, and correctly suggests that the quadratic model is the best fit. In other words, if done correctly then subject-wise-cross-validation will work just fine even for the tiny population chosen by the reviewer. We agree, however, that having too few subjects makes subject-wise CV impossible – and indeed, we do not feel ML papers can tell much about their population performance based on a very small sample.

A **B**

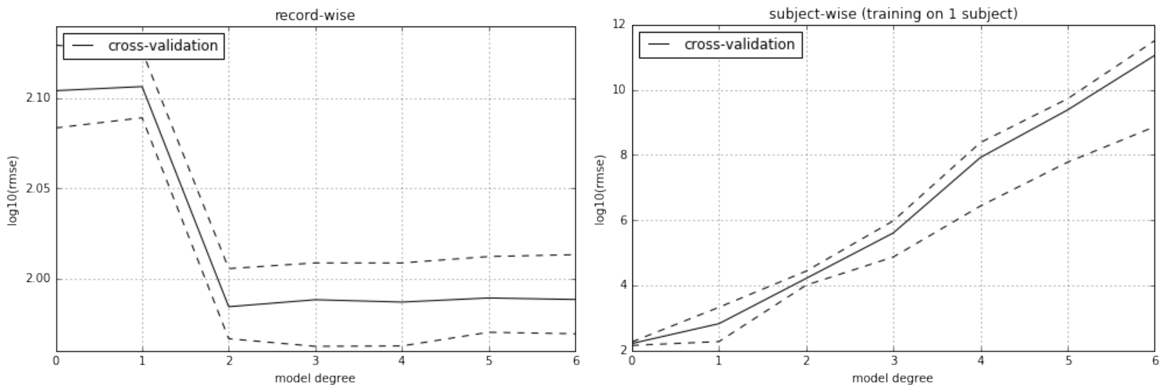

C

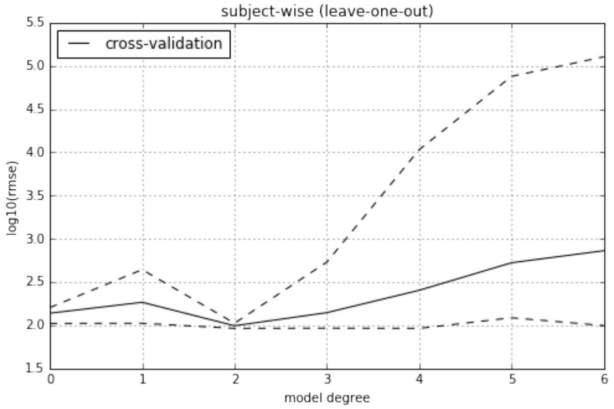

Figure 2. Reproduction of Figure 2.3 simulation in the reviewer's letter. The record-wise model's RMSE (A) shows that the quadratic model is probably the best fit, while the model trained on one subject and tested on the remaining 4 (B) suggests the constant model as the best fit, which is wrong. However, if leave-one-subject-out CV is used (C), the results look different, and the best model suggested by cross-validation is again quadratic.

While these simulations may be enough to show that the results shown in Figure 2.3 of the reviewer's letter are misleading, we also wanted to know how the subject-wise and record-wise RMSEs compared to the true prediction errors here. To calculate the true prediction error, we generated an additional subject using the same generative model, shown by green dots in Fig 3A. Then, we evaluated the prediction error of both subject-wise and record-wise models on this new subject. As the results in Fig 3B and 3C show, both models underestimate the error. However, the record-wise model has a much larger underestimation, and it especially fails at predicting the error for more complex models. Prediction errors estimated using subject-wise CV, on the other hand, are much closer to the true prediction error and worsen as model complexity increases, the same way that the true prediction error does. Again, these biases are exactly as they should be predicted.

A

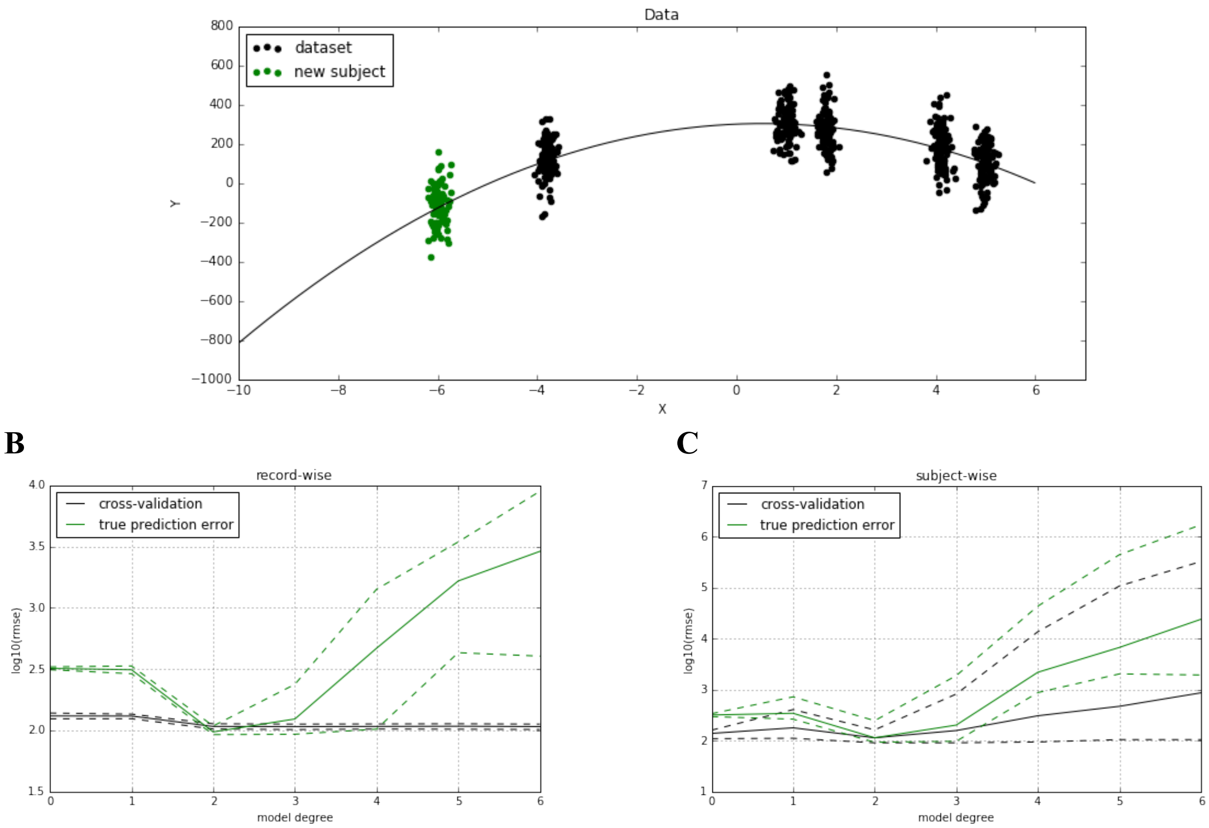

Figure 3. The goal of cross-validation is to estimate the true prediction error, which in this context can be defined as the predicted RMSE on a new subject, shown by green dots (A). While the record-wise CV is massively underestimating this generalization error (B, green), the cross-validation error estimated by subject-wise CV is much closer to the generalization error (C), and it correctly follows its trend over the complexity of the model. Solid lines show the mean, and dashed lines show the minimum and maximum values.

Response to specific arguments and comments

Comment: "But a huge amount is already known and written about this (e.g. read Hand, 2006) and this paper does not get us further towards this goal because of the fundamental misapprehension about the mathematical foundations of CV. Nor does the excessive hyperbole (see specific comments below) and the overblown polemical rant (that belongs on the editorial opinion pages, or Twitter, and not in a scientific article)".

To our knowledge, no one has previously shown the difference between subject-wise and record-wise cross-validation procedures in the way we have shown in this paper. The paper cited above (Hand, 2006) does not discuss the problem of subject-wise versus record-wise cross-validation, therefore, we cannot agree with this comment. Quite specifically, we believe that this is the first paper about subject vs record-wise cross-validation. We believe that it is the first literature analysis in this area, that it is the first paper providing detailed insights into the biases produced by this approach. While we also do not perceive our paper as an "overblown polemical rant", we did tone down the wording in line with the suggestions of both reviewers.

Comment: Figure 2.2. "As can be seen, while the data is drawn i.i.d. from a mixture distribution, the non-stratified (recording-wise) scheme leads to indistinguishable distributions

for training and test sets, whereas, in the stratified (subject-wise) case, the train/test distributions are radically different. Therefore, covariate stratification (i.e. splitting by subject ID) violates an essential assumption of cross-validation.
In the reviewer's simulation (Fig. 2.2), there are only 3 subjects. But generalizing from one subject to the others is almost meaningless and no one should be surprised by the algorithm failing in such a scenario – and no journal should publish ML papers with N=3. This is exactly why we need as many subjects as possible in our dataset. Again, it is not important here how low the cross-validation error is. The importance is how well the algorithms perform on new data, which obviously, given the sample size of 3, must be very bad. This bad performance is truly predicted by a subject-wise CV scheme, as Dr. Little points out, while the use of record-wise CV here would be completely misleading.

Comment: What are the practical limitations of CV, and what are the alternatives? The issues can be subtle and quite complex [Kubota et al., 2016]…. In my opinion, another, similarly valuable direction, is to raise awareness of more appropriate techniques which can be used in the situation where distributional mismatch occurs, e.g. covariate shift [Sugiyama et al., 2007, Shimodaira, 2000], domain adaptation [Blitzer et al., 2008], methods for class imbalance, sample selection bias and dealing with data drift [Hand, 2006].
Domain adaptation is about adapting algorithms to a new dataset with differently-distributed features (covariates). The focus of this paper is on how to train and cross-validate algorithms properly on an existing dataset, with non-shifting covariates. Let us first solve this problem and then we can move on to problems beyond CV – which should be part of another paper.

Title. This doesn't make any sense. What do the authors mean exactly by "voodoo machine learning" and "clinical predictions"? The title needs to be changed to actually describe the topic of the paper.
The title is a nod to a famous preprint by Ed Vul that deals with other statistical mistakes in the literature. Realizing that this link will be lost to much of our audience we decided to delete it.

P2, L36: "however, several cross-validation methods exist and only some of them are statistically meaningful." What exactly is meant by this? Yes, there are many CV methods [Arlot and Celisse, 2010, Shimodaira, 2000, Sugiyama et al., 2007], but they are all, by definition "statistically meaningful". So, this statement needs to be corrected.
We now very clearly define what it means for a statistical procedure to be meaningful for a real world problem at hand. We thus also clarified this sentence.

Simulated Clinical Prediction. This is an interesting experiment but it is not sufficiently well described to allow replication. Are the +1/-1 labels which indicate healthy/disease status generated at random? If so, what is their distribution (e.g. Bernoulli)? If ys,r,n is an S × R × N tensor, how can it be added to bs,nus,n which, I assume is an S ×N matrix? What is the feature data which is used as input to the RF classifier, and what are the corresponding labels?
For each generated dataset, half of the subjects were set to diseased (1) and half were healthy (-1). Lower-case, non-bold symbols represent scalar variables throughout the whole paper, not matrices, and therefore they can be added. We realized that this paragraph could be confusing to the reader, and therefore we revised it in the new version of our paper.
Further comments on wording

Throughout the paper we softened the wording. However, to be clear, we continue to consider record-wise cross-validation when promising generalization to new subjects, not to be a subtle sub-optimality, but a major error that needs to preclude publication in journals. We would be very happy to have this statement be evaluated by professional statisticians (which we informally did).

References

Arlot, S., & Celisse, A. (2010). A survey of cross-validation procedures for model selection. Statistics surveys, 4, 40-79.
Hand, D. J. (2006). Classifier technology and the illusion of progress. Statistical science, 21(1), 1-14.
Rice, J. A., & Silverman, B. W. (1991). Estimating the mean and covariance structure nonparametrically when the data are curves. Journal of the Royal Statistical Society. Series B (Methodological), 233-243.
Xu, G., & Huang, J. Z. (2012). Asymptotic optimality and efficient computation of the leave-subject-out cross-validation. The Annals of Statistics, 40(6), 3003-3030.

Response to Reviewer 2

Comment: When first reading the title one could think that this is a junk paper. After reading the whole paper it turns out to be a serious and very well researched contribution. First hand it is highly recommendable to change the title as the paper does in no way deal with any Voodoo aspects and it is not explained what is meant with Voodoo, so either explain what is Voodoo machine learning or change the title.
We thank the reviewer for the helpful feedback and agree that the title was a bit misleading. Therefore, we changed it to <u>"The need to approximate the use-case in clinical machine learning"</u>. Although we want to add that this was a nod to a well-known preprint by Ed Vul who used the same voodoo verbiage. They also made him take it out as part of the review process.

Comment: The paper is readable and has a good structure; acronyms are also defined, here some suggestions on how to further improve the paper:
Check and revise formatting (whole paper)
We have formatted the paper to comply with the journal requirements. We will be happy to modify that based on specific suggestions of the editor and the reviewer.

Comment: Figures should be placed into the related paragraphs
We agree with the reviewer that it would be more appropriate to place figures into the related paragraphs. However, figures were placed at the end of the manuscript to comply with the journal formatting requirements during submission. We can include the figures in the text as well if the reviewer and the editor suggest. We agree that the world would be a better place if journals would switch to a figures-in-text format for submission.

Comment: Descriptions of the data sets have no references?
The reference to the publicly available human activity recognition dataset [19] has been updated with the correct URL. References to the code for generating the simulated dataset and the systematic review results are in the section "Availability of Supporting Data and Materials", as required by the journal formatting guidelines.

Comment: Please mention the exact dataset(s) that were used in this research.
The database that we used was called "Smartphone-Based Recognition of Human Activities and Postural Transition Data Set". We have now specified the exact name of this dataset in the section "Availability of Supporting data and materials". In addition, we have provided the correct reference and link to the dataset. We also clarified that the generative model code can be downloaded by following the link mentioned in the same section.

Comment: on page 11, criteria 7) it says "Studies for which the CV type was "unknown"" - should this not read "not unknown"?
That is correct. We changed "unknown" to "not unknown".

Comment: Figures 3 and 5 are blurry
We apologize for that. We believe this is a result of the PDF generation process following the upload of the manuscript, which is beyond our control. However, it is possible to download the

original high-resolution figures by clicking on the link on the top of each page. We hope this helps addressing the issue.

Comment: Link to 'dataset' in reference 17 does not work (one "]" too much in URL)
The reference 19 (17 in previous submission) has been now updated with the correct URL.

Comment: Link in reference 17 only refers to the ML Repository of UCI, not a particular dataset

The link has been corrected, and now points to the specific dataset that we used.

Comment: General structure of the paper could be enhanced
We followed the specific format requirements of the journal. We will be happy to modify that based on specific suggestions of the editor and/or the reviewer.

Comment: Do not forget to revise the title (current title is misleading)
We did revise the title.

Comment: On page 3, line 59 you say: "In medicine, a great hope for machine learning is to automatically detect or predict disease ..."
Please mention here that in medicine, particularly in clinical medicine, automatic approaches greatly benefit from big data with many training sets. However, in the health domain, sometimes you are confronted with a small number of data sets, complex data, or rare events, where automatic approaches suffer of insufficient training samples. Here interactive Machine Learning may be of help, see:
Holzinger, A. 2016. Interactive Machine Learning for Health Informatics: When do we need the human-in-the-loop? Springer Brain Informatics, 3, (2), 119-131, doi:10.1007/s40708-016-0042-6, and an experimental example: Holzinger, A., Plass, M., Holzinger, K., Crisan, G., Pintea, C. & Palade, V. 2016. Towards interactive Machine Learning (iML): Applying Ant Colony Algorithms to solve the Traveling Salesman Problem with the Human-in-the-Loop approach. Springer Lecture Notes in Computer Science LNCS 9817. Heidelberg, Berlin, New York: Springer, doi:10.1007/978-3-319-45507-56.
On page 3, line 64-66, here is another recent clinical example of related work:
Donsa, K., Spat, S., Beck, P., Pieber, T. R. & Holzinger, A. 2015. Towards personalization of diabetes therapy using computerized decision support and machine learning: some open problems and challenges. In: Holzinger, A., Roecker, C. & Ziefle, M. (eds.) Smart Health, Lecture Notes in Computer Science LNCS 8700. Heidelberg, Berlin: Springer, pp. 235-260, doi:10.1007/978-3-319-16226-3_10.

We thank the reviewer for the suggested citations. We have included this aspect in the Discussion along with the two suggested citations (second to the last paragraph). We also added (Donsa et al, 2015) as another application to the Introduction (first paragraph).

Comments on the Saeb et al manuscript and review

Gaël Varoquaux

Intervening in a review process is a difficult task, even more so after a thoughtful discussion on subtle points. The authors have one view on the manuscript; the reviewer has a differing one; and I have a third one, also different. I believe that the manuscript is overall correct, but has simplified messages that may have prompted some of the reviewers comments, based on valid considerations. The challenge is finding the right trade off in simplicity: simple messages are more easily understood, but may mislead; subtle messages may not come across. I give below first my take on the exchange between the reviewer and the authors, and in a second step possible ways forwards.

My analysis of the two points of view

My reading on the manuscript: simplified but useful

The topic of the original manuscript is important, and I must praise the authors for trying to draw the community's attention on the important of realistic settings with regards to cross-validation. I like the new title, which is a good message to give.

Indeed, as the manuscript stresses, having data from a given subject in the train and the test set implies that the prediction can rely on some subject-specific information, and hence might be optimistic and not reflect the application settings.

Yet, the manuscript feels a bit restrictive in its considerations, stating "CV in clinical machine learning is thus subject-wise: here the training and test sets contain records from different subjects". On the one hand, in the case of diagnosis applications it is indeed important to split data from subject. However, for prognosis on a subject the relevant split might be along the time dimension. In more complex settings, such as multi-site data, the correct evidence to argue that a given biomarker is useful across clinical sites would be cross-validation across sites, as my group recently demonstrated [1].

As a additional remark, an unfortunate consequence of the simple dichotomy painted by the other between leave-one-record-out and leave-one-subject-out CV is that practitioners will then massively use leave-one-subject-out. It is indeed better than leave-one-record-out for most applications. Yet, cross-validation strategies leaving out single instances (leave *one* out) give higher variance and are more fragile to dependencies across samples than strategies leaving out larger test sets. This point has been known for a very long time in the machine-learning literature. Our recent recent of cross-validation in neuroimaging [2] has touched upon this aspect of cross-validation, as well as others, including the problems raised by this manuscript (biases due to non-independence between train and test), both citing the machine-learning literature and with empirical results.

For these reasons, I agree with Dr. Little's comment on the fact the manuscript touches only the surface of the problem and contributes little to the existing literature on the topic. It is useful from the point of the sociology of research, to emphasize a simple message, but only from this point. Subject-wise versus record-wise cross-validation is nothing more than a special instance of the wider problem of independence between tests and train set. This problems is very well known in data-processing field (it is sometimes referred to as "data snooping"). Narrowing the discussion to the specific case at hand is useful for didactic purposes, but will probably be of limited long-term use in an application field, given that researchers will soon encounter other instances of this problem.

My comments on the dialogue in the review process

I.i.d. assumption

The assumption of i.i.d. data has been a sticking point in the exchange.

Data points do not need to be i.i.d. for CV to be a useful estimate of some generalization score. Mathematical studies of CV tend to make this hypothesis to facilitate theoretical analysis. However, the crucial aspect is the independence of train and test set (as stated in the reply of the authors to the reviewers). If it is of interest, our review on cross-validation in neuroimaging [2] discusses how theses aspects come into play in biomarker applications, with possibly multiple subjects and confounding factors.

The discussion between the authors and the reviewer on i.i.d. properties is, in my opinion, difficult to lead without agreement on the variables under study and their probabilistic model. Typically, a model for multisubject data would be a 2-level hierarchical model. In this sense, data may be i.i.d. for a given subject; the subject-to-group residual errors may be i.i.d.; and the matrix of data describing a subject may be i.i.d; but the individual samples would not be i.i.d. across subjects. This model is close to that proposed by Dr Little in section 4 of his review, but the variable a would then described as a random variable, i.i.d. over k. Whether or not the data partitioning that k creates is over interest to the prediction settings depend on the application (see below). From a theoretical perspective, to fit in the analysis of Dr. Little, the different scenarios correspond to whether the estimate of predictive power that cross-validation performs is taken marginally or conditionnally upon the confounding variable Z. Hence both the position of the reviewer and that of the author may be valid, depending on what exact application is considered. That said, this theoretical discussion is maybe beyond the scope of the present manuscript, not only because it may not be formulated in a way that practitioners are used to, but also because the manuscript is currently focused --for the better or the worst-- on a specific setting and a specific misinterpretation.

The review argues that data differ across subjects, backing this point with figure 2.1. This is true, subjects are not identical. Yet, i.i.d. assumption across subjects may simply mean that this difference across subjects is drawn randomly and independently from the same distribution in all subjects. Such an assumption may be broken if there are sub-groups in subjects, for instance when there are patients and controls, or when the subjects are drawn from different sites [1]. As we explain in [2], the importance is then to preserve independence between test and train. That said, to put this discussion in the context of the present manuscript, different cross-validation strategies should be applied depending on the application settings. If the goal is to measure predictive power of a model across acquisition sites, full sites must be left out. But, if the goal is to predict a diagnostic status independently of age or movement, the test and train set should be balanced in terms of both the confounding variable --age or movement-- and the clinical variable of interest.

Finally, Dr Little raises and interesting point about cross-validation: "this procedure only works if we are testing and training on the same distribution, otherwise the model cannot be expected to make meaningful predictions on the testing data". The point is partly valid, in the sense that non i.i.d. properties of the data can easily break predictive models. However, cross-validation can be used to measure their predictive power in these settings, and the fragility of models implies that such measure is important to establish the validity and usefulness of prediction in the application settings.

What does cross-validation measure?

In non i.i.d. settings, the question of the quantity measured by cross-validation is more subtle.

Cross-validation to new records or to new subjects does not measure the same thing. Dr Little's point of view is that "the authors have uncovered [...] good evidence that the use of subject-wise CV overestimates the prediction error, rather than evidence that recording-wise CV underestimates". I would argue that these two approaches measure a different things, but that the measure given by cross-validation across subjects is more useful than cross-validation including common records and that it is an over-estimation of the predictive power in most application settings.

From an application standpoint, most often the goal is some form of diagnosis across subjects, hence the cross-validation should be done across subjects. However, if the goal is prognosis for a given subject, the practitioner would have access to a subject's present data and try to predict a future state. The experimental procedure to measure the success of a given data-processing pipeline would then be much more subtle, as it would have to mimic application settings while avoiding data snooping.

On the other hand, in basic research such as cognitive neuroimaging, cross-validation results are often used to claim evidence for shared information between two sources of data, for instance imaging and behavioral. In these settings, there are no important application goals to predict across subjects. Hence,

one might think that cross-validation with multiple subjects and shared records in train and test set might give interesting results. This is not the case if the variable to be predicted, eg the behavioral variable, differs across subjects. Indeed, prediction of this variable might then be based on the identification of the subject, and hence not demonstrate any link between the variables of interest. This aspect is mentioned in the manuscript L92, page 4.

My recommendations

Where to go now? There are several options: the authors could use the feedback of the review process, including my comment above, to change their manuscript. Such changes might give a different flavor to the manuscript than the one that they are willing to endorse. Curated reviews might be published jointly with the manuscript, including a rewritten version of my comments. These could either be compiled in one commentary, cosigned by reviewers, namely Dr Little and myself, provided we can reach an agreement, or appear as two commentaries. They may or may not be commented upon and endorsed by the original authors. Ideally, the whole set of works would be published together, in order to give readers a good view on the various facets of the problem.

References

As this is a non-anonymous informal review, I am citing only my own work in the review. Indeed, the other major references that comes to my mind have already been brought up in the discussion, and in the interest of time I am not performing a literature review; merely pointing to works that I understand well.

[1]: Abraham et al, "Deriving robust biomarkers from multi-site resting-state data: An Autism-based example", BioRxiv, http://biorxiv.org/content/early/2016/09/19/075853.abstract (under major revisions)

[2]: Varoquaux et al, "Assessing and tuning brain decoders: cross-validation, caveats, and guidelines", ArXiv, https://arxiv.org/abs/1606.05201 (under minor revision)

Responses to Dr. Varoquaux's Comments

We are very thankful for the careful and constructive reading of our paper by the reviewer. We agree on essentially all aspects of the reviewer's analysis. We extensively rewrote the manuscript in response to the comments. Most specifically, we toned down some inflammatory language (although we still, just among us, believe that many clinicians are misguided). We also improved the discussion of scenarios for cross-validation. Lastly, we agree that among machine learning experts the issue is well known. However, the field needs to hear about this problem and a lot of real machine learning experts give up on the field (personal communications) because they can, using the correct techniques, not replicate such high accuracy values as the field often erroneously reports. As such, we think that for this audience this is important. In any case, we are indebted to the careful review.
Below, the reviewer's comment are in *italic*, and the responses in regular format.

My reading on the manuscript: simplified but useful
The topic of the original manuscript is important, and I must praise the authors for trying to draw the community's attention on the important of realistic settings with regards to cross-validation. I like the new title, which is a good message to give. Indeed, as the manuscript stresses, having data from a given subject in the train and the test set implies that the prediction can rely on some subject-specific information, and hence might be optimistic and not reflect the application settings.
We are happy that this message got across!

Yet, the manuscript feels a bit restrictive in its considerations, stating "CV in clinical machine learning is thus subject-wise: here the training and test sets contain records from different subjects". On the one hand, in the case of diagnosis applications it is indeed important to split data from subject. However, for prognosis on a subject the relevant split might be along the time dimension. In more complex settings, such as multi-site data, the correct evidence to argue that a given biomarker is useful across clinical sites would be cross-validation across sites, as my group recently demonstrated [1].
We agree, and as we have mentioned both in the manuscript and in our response to Dr. Little's comments, here we are focusing only on global models, which are trained to estimate clinical states across the subjects (diagnosis). We do not deal with personal models which estimate clinical states of the same subject at a different time (prognosis). This is why we have excluded the studies that have used personal models – see Eligibility Criteria, item #5. However, we understand that the paper, especially in the Introduction, was not very clear on this point. Therefore, we have revised the Introduction and included an additional paragraph on what form of cross-validation should be used for each scenario. We also included the cross-site cross-validation scenario suggested by the reviewer as another possible scenario, along with the suggested reference.

As a additional remark, an unfortunate consequence of the simple dichotomy painted by the other between leave-one-record-out and leave-one-subject-out CV is that practitioners will then massively use leave-one-subject-out. It is indeed better than leave-one-record-out for most applications. Yet, cross-validation strategies leaving out single instances (leave one out) give higher variance and are more fragile to dependencies across samples than strategies leaving out

larger test sets. This point has been known for a very long time in the machine-learning literature. Our recent recent of cross-validation in neuroimaging [2] has touched upon this aspect of cross-validation, as well as others, including the problems raised by this manuscript (biases due to non-independence between train and test), both citing the machine-learning literature and with empirical results.
The focus of the paper is on subject-wise versus record-wise cross-validation, and leave-one-subject/record-out is only a special case of these. Decreasing the size of the cross-validation folds, in the extreme case to only one sample, indeed increases the variance of estimating the prediction error, and therefore choosing the proper fold size has been the subject of previous studies (see [1] for an example). In this paper, we did not focus on choosing the fold size, neither we considered the special case of leave-one-out. We modified the Introduction, and also modified Figure 1B so that it is showing a general picture of record-wise CV rather than the special case of leave-one-record-out.

For these reasons, I agree with Dr. Little's comment on the fact the manuscript touches only the surface of the problem and contributes little to the existing literature on the topic. It is useful from the point of the sociology of research, to emphasize a simple message, but only from this point. Subject-wise versus record-wise cross-validation is nothing more than a special instance of the wider problem of independence between tests and train set. This problems is very well known in data-processing field (it is sometimes referred to as "data snooping"). Narrowing the discussion to the specific case at hand is useful for didactic purposes, but will probably be of limited long-term use in an application field, given that researchers will soon encounter other instances of this problem.
We agree that the subject-wise versus record-wise dichotomy is a special case of the problem of independence between train and test sets in machine learning. However, we cannot agree with the comment that it is just a special instance in the clinical prediction domain which is the focus of our study. The fact that we have found almost half of the literature to use cross-validation in an inappropriate way is enough to show that this very obvious fact has been ignored by many researchers. We also believe that these results will be of long-term use, because within-subject versus cross-subject variability is a fundamental fact in human subject data. We hope that more machine learning researchers will be reviewing papers in this field in the future.

My comments on the dialogue in the review process
I.i.d. assumption
The assumption of i.i.d. data has been a sticking point in the exchange.
Data points do not need to be i.i.d. for CV to be a useful estimate of some generalization score. Mathematical studies of CV tend to make this hypothesis to facilitate theoretical analysis. However, the crucial aspect is the independence of train and test set (as stated in the reply of the authors to the reviewers). If it is of interest, our review on cross-validation in neuroimaging [2] discusses how theses aspects come into play in biomarker applications, with possibly multiple subjects and confounding factors.
We agree that data points do not need to be i.i.d. for CV to be valid. Therefore, we also believe that the i.i.d. discussion, brought up by Dr. Little, is not helpful here.

The discussion between the authors and the reviewer on i.i.d. properties is, in my opinion, difficult to lead without agreement on the variables under study and their probabilistic model.

Typically, a model for multisubject data would be a 2-level hierarchical model. In this sense, data may be i.i.d. for a given subject; the subject-to-group residual errors may be i.i.d.; and the matrix of data describing a subject may be i.i.d; but the individual samples would not be i.i.d. across subjects. This model is close to that proposed by Dr Little in section 4 of his review, but the variable a would then described as a random variable, i.i.d. over k. Whether or not the data partitioning that k creates is over interest to the prediction settings depend on the application (see below). From a theoretical perspective, to fit in the analysis of Dr. Little, the different scenarios correspond to whether the estimate of predictive power that cross-validation performs is taken marginally or conditionnally upon the confounding variable Z. Hence both the position of the reviewer and that of the author may be valid, depending on what exact application is considered. That said, this theoretical discussion is maybe beyond the scope of the present manuscript, not only because it may not be formulated in a way that practitioners are used to, but also because the manuscript is currently focused --for the better or the worst-- on a specific setting and a specific misinterpretation.
We cannot agree more. But we want to emphasize again that the focus of this paper is on global models trained for diagnosis, where the goal is to estimate the generalizability of the model to new subjects. The scenario shown in Figure 2.3 of Dr. Little's comments, although not accurate as we showed in our last response letter, is not relevant here as its goal is model selection.

The review argues that data differ across subjects, backing this point with figure 2.1. This is true, subjects are not identical. Yet, i.i.d. assumption across subjects may simply mean that this difference across subjects is drawn randomly and independently from the same distribution in all subjects. Such an assumption may be broken if there are sub-groups in subjects, for instance when there are patients and controls, or when the subjects are drawn from different sites [1]. As we explain in [2], the importance is then to preserve independence between test and train. That said, to put this discussion in the context of the present manuscript, different cross-validation strategies should be applied depending on the application settings. If the goal is to measure predictive power of a model across acquisition sites, full sites must be left out. But, if the goal is to predict a diagnostic status independently of age or movement, the test and train set should be balanced in terms of both the confounding variable --age or movement—and the clinical variable of interest.
We agree that the importance is preserving the independence. We have clearly mentioned this in the revised manuscript (L85). In our application scenario, which is global clinical predictions, preservation of independence means that full subject records must be left out for cross-validation. This point is also now clearly mentioned in the Introduction.

Finally, Dr Little raises and interesting point about cross-validation: "this procedure only works if we are testing and training on the same distribution, otherwise the model cannot be expected to make meaningful predictions on the testing data". The point is partly valid, in the sense that non i.i.d. properties of the data can easily break predictive models. However, cross-validation can be used to measure their predictive power in these settings, and the fragility of models implies that such measure is important to establish the validity and usefulness of prediction in the application settings.
This is a very valid point. Cross-validation should be determined by the application scenario, not by the statistics of the data. The fact that, for a given model, an appropriately-chosen cross-

validation may result in poor prediction performance implies that the model's prediction power is limited, not that the cross-validation method is wrong.

What does cross-validation measure?

In non i.i.d. settings, the question of the quantity measured by cross-validation is more subtle. Cross-validation to new records or to new subjects does not measure the same thing. Dr Little's point of view is that "the authors have uncovered [...] good evidence that the use of subject-wise CV overestimates the prediction error, rather than evidence that recording-wise CV underestimates". I would argue that these two approaches measure a different things, but that the measure given by cross-validation across subjects is more useful than cross-validation including common records and that it is an over-estimation of the predictive power in most application settings.

From an application standpoint, most often the goal is some form of diagnosis across subjects, hence the cross-validation should be done across subjects. However, if the goal is prognosis for a given subject, the practitioner would have access to a subject's present data and try to predict a future state. The experimental procedure to measure the success of a given data-processing pipeline would then be much more subtle, as it would have to mimic application settings while avoiding data snooping.

Again, the focus of this paper is on diagnosis across the subjects, not prognosis. Therefore, as the reviewer has mentioned, the cross-validation must be subject-wise. We believe that the revised manuscript has made this point clear to the reader.

On the other hand, in basic research such as cognitive neuroimaging, cross-validation results are often used to claim evidence for shared information between two sources of data, for instance imaging and behavioral. In these settings, there are no important application goals to predict across subjects. Hence, one might think that cross-validation with multiple subjects and shared records in train and test set might give interesting results. This is not the case if the variable to be predicted, eg the behavioral variable, differs across subjects. Indeed, prediction of this variable might then be based on the identification of the subject, and hence not demonstrate any link between the variables of interest. This aspect is mentioned in the manuscript L92, page 4.

We agree that in some applications, such as in neuroimaging, the goal is not necessarily to generalize across the subjects. That is why we limited our literature search to clinical application studies which use global models, and generally claim to have developed diagnosis/detection algorithms that can be used for new subjects.

My recommendations

Where to go now? There are several options: the authors could use the feedback of the review process, including my comment above, to change their manuscript. Such changes might give a different flavor to the manuscript than the one that they are willing to endorse. Curated reviews might be published jointly with the manuscript, including a rewritten version of my comments. These could either be compiled in one commentary, cosigned by reviewers, namely Dr Little and myself, provided we can reach an agreement, or appear as two commentaries. They may or may not be commented upon and endorsed by the original authors. Ideally, the whole set of works would be published together, in order to give readers a good view on the various facets of the problem.

We have made changes in the manuscript according to the reviewers' comments. Specifically, we have softened the language, and we have revised the manuscript so that the reader clearly understands which application scenarios are the focus of this paper. However, we are also open to have Dr. Varoquaux's and Dr. Little's comments (maybe minus the misleading i.i.d. discussion) published as commentary alongside the paper.

References

[1] Kohavi, Ron. "A study of cross-validation and bootstrap for accuracy estimation and model selection." Ijcai. Vol. 14. No. 2. 1995.
