# Supplementary material for: The need to approximate the use-case in clinical machine learning
Source: Gigascience. 2017 Mar 15;6(5):1–9. doi: 10.1093/gigascience/gix019 (PMC5441397; doi:10.1093/gigascience/gix019)
Supplement: Response_to_reviewer_comments_Original_Submission.pdf [file gix019_Response_to_reviewer_comments_Original_Submission.pdf]

The response letter is attached as supplementary material.
